# Clinical outcomes and quality of life after proton therapy for cranial and extracranial solitary fibrous tumors

**DOI:** 10.1093/bjro/tzag023

**Published:** 2026-10-26

**Authors:** Philipp Reinhardt, Alessandra Bolsi, Damien Charles Weber

**Affiliations:** Center for Proton Therapy, Paul Scherrer Institute, ETH Domain, 5232 Villigen, Switzerland; Department of Radiation Oncology, Inselspital, Bern University Hospital, University of Bern, 3012 Bern, Switzerland; Center for Proton Therapy, Paul Scherrer Institute, ETH Domain, 5232 Villigen, Switzerland; Center for Proton Therapy, Paul Scherrer Institute, ETH Domain, 5232 Villigen, Switzerland; Department of Radiation Oncology, Inselspital, Bern University Hospital, University of Bern, 3012 Bern, Switzerland

**Keywords:** solitary fibrous tumor, brain tumor, spinal tumor, proton therapy, pencil beam scanning, patterns of failure, quality of life

## Abstract

**Objectives:**

Solitary fibrous tumors (SFTs) are rare malignancies. Surgical resection, particularly gross total resection, is the mainstay of therapy. The benefit of postoperative radiotherapy (RT) remains uncertain, with inconsistent findings across studies. Existing literature predominantly reported photon-based RT. This study evaluates clinical outcomes, late toxicities, and quality of life (QoL) in patients with SFTs treated with postoperative pencil beam scanning proton therapy (PT).

**Methods:**

We performed a retrospective single-center analysis of patients with histologically confirmed cranial or extracranial SFTs treated with PT. Local control (LC) was assessed using Brain Tumor Reporting and Data System (BT-RADS) or Response Evaluation Criteria in Solid Tumors (RECIST) criteria. Toxicities were recorded using Common Terminology Criteria for Adverse Events (CTCAE) v5.0, and QoL was assessed prospectively in patients with cranial SFTs. Kaplan-Meier analysis was used to estimate overall survival (OS) and LC, with subgroup comparisons made using the log-rank test.

**Results:**

Seventeen patients were included, with a majority presenting with cranial SFTs (64.7%). After a median follow-up of 47 months (range 7-208 months), OS was 100%. The 5-year LC rate was 81.8% (95% CI, 61.9%-100%) for the entire cohort. Acute toxicities were limited to grade 1-2. Two patients (11.8%) experienced severe late toxicity. Overall QoL appeared broadly stable over time and was comparable to reference population values.

**Conclusion:**

PT provided encouraging preliminary OS and LC rates in patients with cranial and extracranial SFTs. Despite a low rate, the severity of these late toxicities highlights substantial risks near the central nervous system axis.

**Advances in knowledge:**

This retrospective single-center study provides the largest analyzed cohort of patients with cranial and extracranial SFTs treated with PT to our knowledge.

## Introduction

Solitary fibrous tumors (SFTs) are uncommon fibroblastic neoplasms that represent <1% of all primary central nervous system (CNS) tumors. There is limited evidence to guide specific treatment recommendations due to their rarity. Surgical resection, particularly gross total resection (GTR), remains the cornerstone of treatment. However, the benefit of postoperative radiotherapy (PORT) remains debated, with studies showing varying results. A recent systematic review by Na et al provided evidence supporting the use of PORT following GTR.^1^ The authors reported improved progression-free survival (PFS) and overall survival (OS) among patients with intracranial SFTs. These results contrast with earlier reports that did not clearly demonstrate a survival advantage with PORT in this setting.^2–4^ Despite the potential clinical benefit of PORT, only limited evidence exists for the role of pencil beam scanning proton therapy (PT). Additionally, data on toxicity and quality of life (QoL) after PORT are largely lacking.

Particle therapy has emerged as a promising modality due to its favorable dose distribution and potential to spare surrounding healthy tissues, which is an important consideration in anatomically complex regions such as the skull base or spine. However, the evidence for particle therapy in SFT is limited to case reports and small series, often within heterogeneous patient cohorts treated with various radiotherapy (RT) modalities. Recently, Ton et al^5^ reported the results of 29 patients with intracranial SFTs treated with PORT, among whom 1 was treated with carbon ions and 10 were treated with protons. The authors demonstrated promising results with 5-year PFS rates of 86% and 5-year local PFS of 96%. However, no subgroup analysis was performed regarding particle therapy.

In addition to efficacy, treatment-related toxicity, and patient-centered outcomes such as QoL are crucial considerations in the management of SFTs. However, data on toxicity after PORT remain limited, and only a few studies have prospectively assessed QoL in this rare disease. Early reports suggest that PORT is generally well tolerated, but robust evidence on long-term toxicity and QoL is still lacking.

The present retrospective analysis addressed this knowledge gap by reporting on the largest single-center cohort of patients with cranial and extracranial SFTs treated exclusively with PT. Our study aims to report the oncological outcomes, evaluate toxicity, and assess its impact on QoL.

## Methods

### Patient population

We performed a retrospective, single-center analysis of patients diagnosed with an SFT. In total, 17 patients with histologically confirmed SFT located either cranially (*n* = 11) or extracranially (*n* = 6) were included in the study. All patients underwent pencil beam scanning PT between January 2002 and December 2024. Histopathological diagnosis and grading were performed according to the World Health Organization (WHO) CNS classification in use at the time of diagnosis.^6–8^ Because the study period spanned several changes in SFT terminology and grading, classification was based on the diagnostic standards applicable at the time of diagnosis. STAT6 positivity was documented in 11 patients, while STAT6 status was unknown in 6 patients. World Health Organization grade was available in 11 patients and unknown in 6 patients. No comprehensive centralized histopathological or molecular reclassification was performed because of the retrospective design and limited availability of archival tissue. In particular, postoperative PT was indicated for patients following subtotal resection (STR). For individuals who achieved a GTR, adjuvant PT was selectively performed for high-risk patients, specifically those with WHO grade 2 or 3 tumors or recurrent disease. Furthermore, PT was frequently utilized due to tumor proximity to organs at risk (OAR). This retrospective study was approved by the local Ethics Committee.

### Treatment planning

Patient immobilization and simulation were tailored to tumor localization, utilizing bite blocks (*n* = 9) or thermoplastic head masks (*n* = 2) for cranial cases, and vacuum immobilization for extracranial cases. The gross tumor volume (GTV) encompassed the macroscopic residual tumor or, in cases of GTR, the corresponding resection cavity. The clinical target volume (CTV) was defined by adding a margin of 3-20 mm to the GTV to account for potential microscopic disease spread. To create the planning target volume (PTV), an additional margin of 4-10 mm was added to the CTV to compensate for setup and range uncertainties. The variation in PTV margins reflects differences in tumor localization and advancements in image-guided equipment and techniques. Field arrangements comprising 2-4 fields were selected according to target location. A relative biological effectiveness (RBE) of 1.1 was assumed, in line with internationally accepted clinical standards. Institutional dose constraints for organs at risk were applied in line with published QUANTEC recommendations and EPTN consensus guidance for CNS normal tissue tolerance.^9^^,^^10^ Under conventional fractionation, the spinal cord was constrained to D0.03 mL ≤ 54 Gy (RBE) at the spinal cord surface and D0.03 mL ≤ 50 Gy (RBE) at the spinal cord center. The same institutional constraints were applied to the brainstem surface and brainstem center, respectively. In re-irradiation cases, individualized plan-by-plan compromises were required. Beyond physical dose constraints, beam arrangements were selected to avoid multiple beams within the same plan terminating in critical OARs, thereby minimizing the accumulation of regions with elevated biological effectiveness in these structures.

### Follow-up

Clinical and radiological data were collected through routine procedures, communication with referring physicians, and patient-reported outcome questionnaires. These data were available through the clinical information system. Most patients underwent follow-up (FU) examinations at their respective referring clinics. To ensure consistency in response assessments, all external FU images were re-reviewed during our regular internal clinical review meetings using standardized Brain Tumor Reporting and Data System (BT-RADS) or Response Evaluation Criteria in Solid Tumors (RECIST) criteria. Furthermore, any documented toxicities were adjudicated during these meetings to confirm their exact Common Terminology Criteria for Adverse Event (CTCAE) grade, relationship to the prescribed PT dose distribution, and likelihood of being treatment related. For patients with intracranial SFT, outcome evaluation was based on the BT-RADS score.^11^ For those treated for extracranial SFT, outcome assessment followed the RECIST criteria.^12^ Local failure (LF) and distant failure (DF) were defined as the time between the start of PT and the first imaging that indicated recurrence. Failure patterns were evaluated based on the extent to which the recurrent tumor overlapped with the high-dose region. Consequently, the high-dose area was defined as the volume that received at least 95% of the total prescribed dose. Central recurrences were classified in cases where more than 95% of the recurrence occurred inside the high-dose area. In cases where more than 80% of the recurrence was located within the high-dose area, the failure was classified as in-field. Marginal failures were defined by 20%-80% of the recurrent tumor volume being within the high-dose area. Lastly, out-of-field recurrences were characterized in cases where the recurrent tumor volume was <20% within the high-dose area.

### Toxicity assessment

Acute toxicity was defined as adverse events occurring within the period of PT and within 90 days after the end of treatment, while late toxicity referred to events manifesting beyond this period. All toxicities were graded using the National Cancer Institute’s CTCAE, version 5.0, which categorizes severity on a scale from grade 1 (mild) to grade 5 (death related to toxicity). Cases presenting with complex or severe toxicities were reviewed and discussed by the clinical team during dedicated internal meetings.

### QoL data (cranial SFT only)

Quality of life data were prospectively collected from patients with cranial SFTs using the EORTC QLQ-C30 and BN20 questionnaires starting in 2015. This validated instrument, designed to assess QoL in cancer patients, was administered prior to, during, and at the completion of PT, as well as annually thereafter. Scoring was performed in accordance with the EORTC manual. Descriptive analyses were conducted, with mean scores reported across all time points from baseline (pre-PT) up to 3 years post-treatment. Scores ranged from 0 to 100, with higher scores indicating better functioning or greater symptom burden, depending on the subscale. Selected items of the C30 questionnaire were compared to reference values from the general European population.^13^ Missing individual items or incomplete questionnaires were addressed using available-case analyses without formal statistical imputation.

### Statistical analysis

Descriptive statistics were used to summarize patient demographics. Categorical variables were reported as frequencies and percentages, while continuous variables were presented as medians with range. Acute and late toxicity grades were assessed separately and reported descriptively. Quality of life data were analyzed by calculating mean scores and SDs at each assessment time point.

Time-to-event analyses were performed for OS, local control (LC), and DF with time calculated from the first day of PT using Kaplan-Meier estimates. Events were defined as follows: death for OS, LF for LC, and distant metastasis for DF. Patients without an event at last FU were censored at the date of last contact.

Univariate survival analysis using the log-rank test was performed to evaluate potential associations between LC and clinical, tumor-related, or treatment-related parameters. All statistical analyses were performed using R software, version 4.3.2. A 2-sided *P*-value <.05 was considered statistically significant.

Quality of life outcomes were assessed descriptively and in an exploratory manner because of the very limited sample size. No formal longitudinal hypothesis testing or comparative statistical analyses were conducted.

## Results

### Patient characteristics

Seventeen patients were included in this retrospective study. The majority were male (*n* = 11, 64.7%), and the median age at diagnosis was 35.5 years (range: 10.9-62.6 years). Previous photon RT had been performed in only 2 patients (11.8%), while 15 patients (88.2%) had not received any previous RT. For the 2 patients who received previous photon RT, treatment was delivered as a salvage re-irradiation for local disease recurrence. Treatment planning for these cases incorporated a meticulous review of prior radiation fields. Cumulative doses to OARs were strictly limited using composite dose–volume histograms to minimize compounding overlapping tissue damage. Proton therapy was performed in the primary adjuvant setting in 9 patients (52.9%), while the remaining 8 patients (47.1%) were treated for a recurrence. Additional patient characteristics are presented in Table 1.

**Table 1:** Patient characteristics and treatment parameters (*n* = 17).

Variable	Value
**Gender, *n* (%)**	
** Female**	6 (35.3)
** Male**	11 (64.7)
**Age at diagnosis (years), median (range)**	35.5 (10.9-62.6)
**Age at start of treatment (years), median (range)**	36.8 (11.3-78.8)
**Salvage re-irradiation, *n* (%)**	
** Yes**	2 (11.8)
** No**	15 (88.2)
**Tumor localization, *n* (%)**	
** Cranial**	11 (64.7)
** Extracranial**	6 (35.3)
**Resection status**	
** Gross tumor resection**	8 (47.1)
** STR**	9 (52.9)
**STAT6 status, *n* (%)**	
** Positive**	11 (64.7)
** Unknown**	6 (35.3)
**WHO grade, *n* (%)**	
** Grade 1**	2 (11.8)
** Grade 2**	4 (23.5)
** Grade 3**	5 (29.4)
** Unknown**	6 (35.3)
**PT timing, *n* (%)**	
** Primary**	9 (52.9)
** Recurrence**	8 (47.1)
**PT with boost, *n* (%)**	
** Yes**	9 (52.9)
** No**	8 (47.1)
**PT fractional dose, Gy (RBE), median (range)**	1.8 (1.8-2.0)
**Total dose, Gy (RBE), median (range)**	59.4 (45.0-74.0)
**PTV volume, mL, median (range)**	92.5 (2-594.7)
**PTV coverage (D95%) (%), median (range)**	95.2 (85.0-99.4)
**FU duration (months), median (range)**	47 (7-208)

Abbreviations: FU = Follow-up; PT = Pencil beam scanning proton therapy; PTV = planning target volume; STR = subtotal resection.

### LC and survival outcome

After a median FU of 47 months (range: 7-208 months), OS was 100%. Local failure occurred in 3 (17%) patients—2 with cranial SFTs and 1 with an extracranial SFT. Among the cranial LFs, 1 occurred out-of-field after 39 months, while the other developed in-field after 64 months. The extracranial SFT recurred in-field after 38 months. The preliminary estimated 5-year LC rate for the entire cohort was 81.8% (95% CI, 61.9%-100%; Figure 1A). When stratified by tumor location, the 5-year LC rate was 80.0% (95% CI, 51.6%-100%) for patients with cranial SFTs and 83.3% (95% CI, 58.3%-100%) for those with extracranial SFTs (*P* = .582).

**Figure 1:**
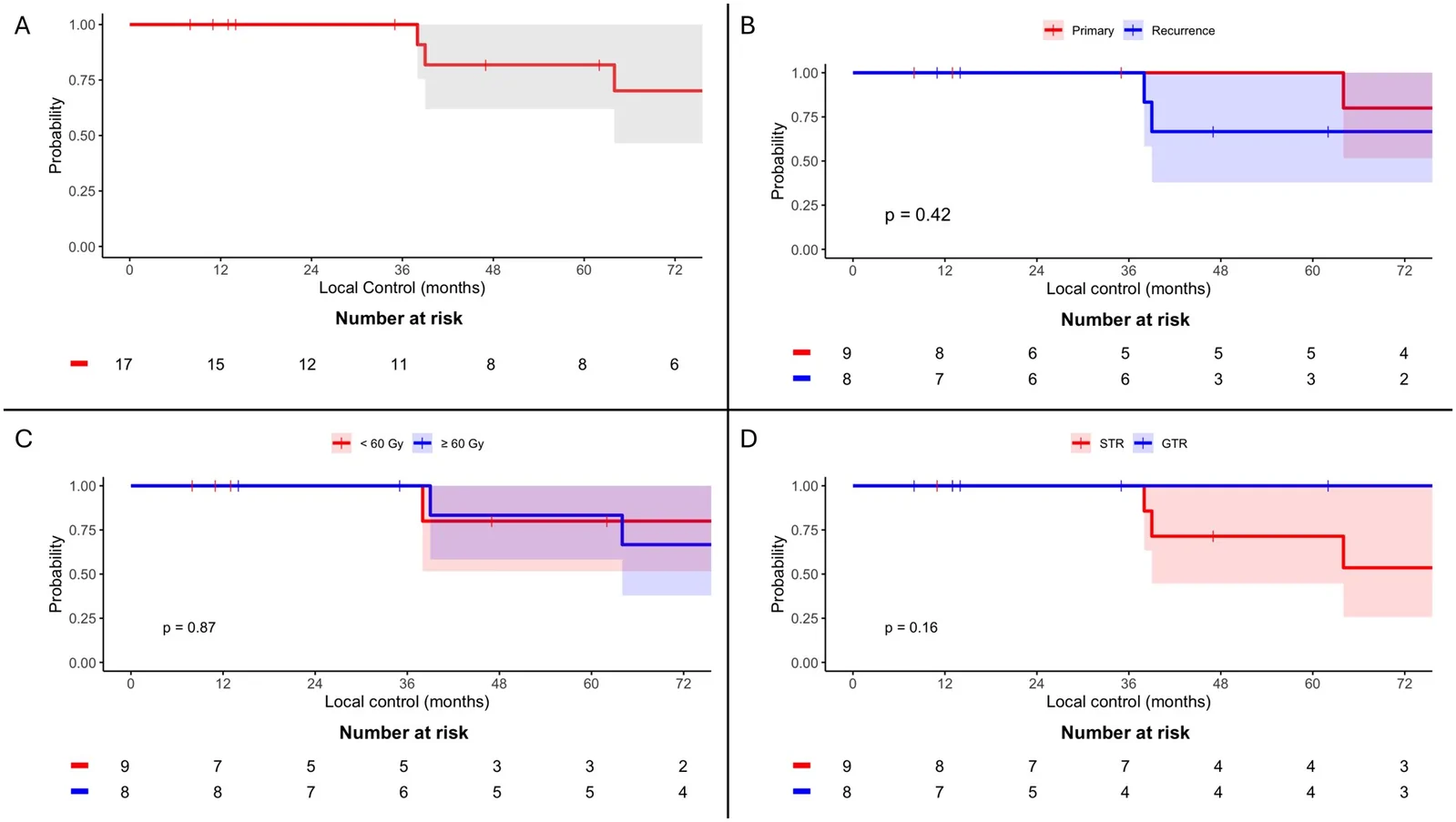
Kaplan-Meier curves for s in the overall cohort (A), and stratified by PT timing (B), total dose (C), and extent of resection (D). Abbreviations: GTR, gross tumor resection; STR, subtotal resection.

There was no significant difference in LC between patients treated in the primary setting versus those treated for recurrence (*P* = .425; Figure 1B), between those who received a total PT dose of <60 Gy (RBE) compared to ≥60 Gy (RBE) (*P* = .866; Figure 1C), or between patients who underwent STR vs GTR (*P* = .159; Figure 1D). Despite the lack of statistical significance, Figure 1D demonstrates a clear trend in LC favoring GTR. To further address cohort heterogeneity, a multicategorical stratification was performed across 4 distinct subgroups (Cranial-Primary, Cranial-Recurrent, Extracranial-Primary, and Extracranial-Recurrent); a log-rank comparison demonstrated no statistically significant divergence in LC behaviors among these combinations (*P* = .76, Figure S1). Overall, univariate analysis showed no statistically significant differences in LC for any of the analyzed parameters (Table 2).

**Table 2:** Univariate analysis for local tumor control (log-rank test).

Variable	Groups compared	Sample size	*P*-value
**Age at diagnosis**	<60 vs ≥ 60 years	14/3	.378
**Gender**	Male vs female	11/6	.866
**Total dose (in high-dose PTV)**	<60 vs ≥60 Gy (RBE)	9/8	.866
**Extent of resection**	STR vs GTR	9/8	.159
**Tumor location**	Cranial vs etracranial	11/6	.582
**PT timing**	Primary vs recurrence	9/8	425
**WHO grade**	1 vs 2 vs 3	2/4/5	.333

Abbreviations: GTR = gross total resection; PT = pencil beam scanning proton therapy; PTV = planning target volume; STR = subtotal resection; WHO = World Health Organization.

A patient-specific timeline illustrates oncological events following diagnosis until disease progression or the last available FU (Figure 2). Surgery was the most common treatment modality. Nine patients underwent a single surgical resection, while the remaining 8 patients had multiple surgeries (mean: 3.88, SD: 1.25). Only 2 patients received concomitant systemic treatment (temozolomide and ifosfamide/doxorubicin). Median time to PT was 5 months with a large range of 1-295 months. Distant metastases occurred in 2 patients (11.8%), both of whom also had an LF. In 1 patient, lung metastases were identified 38 months after PT. The second patient developed a paravertebral soft-tissue metastasis after 13 months and multiple lung and abdominal metastases at 39 months post-treatment.

**Figure 2:**
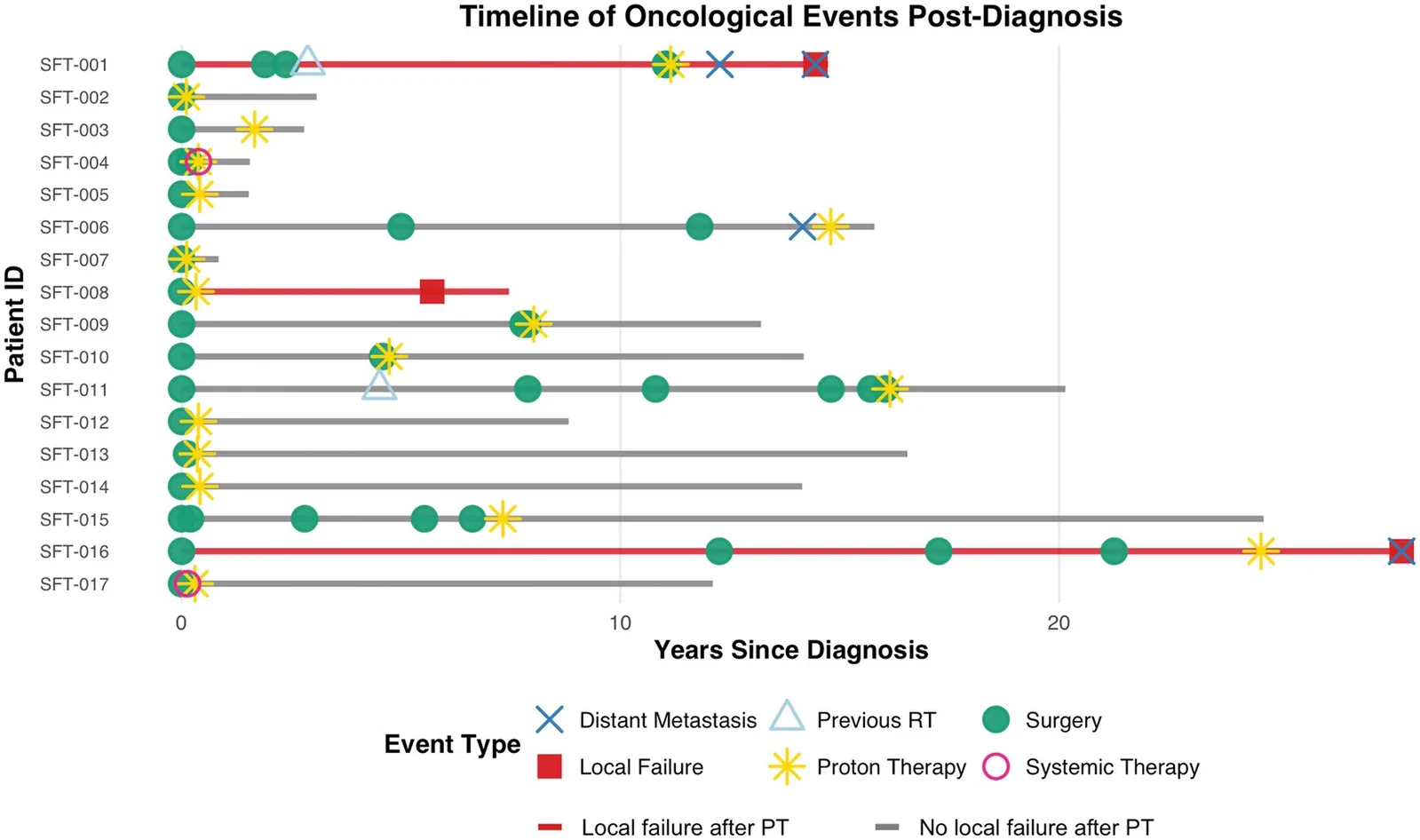
Patient-specific timelines starting from the date of diagnosis. Abbreviations: PT, pencil beam scanning proton therapy; RT, Radiotherapy.

### Toxicity

PT was well tolerated, with all patients experiencing only grade 1 or 2 acute side effects. The most commonly observed acute toxicities included fatigue, dermatitis, and alopecia (Table 3). No acute toxicity grade ≥3 was reported. Late toxicity of grade ≥3 was observed in 2 out of 17 patients (11.8%). Notably, both patients who underwent re-irradiation experienced no high-grade late toxicity. One patient underwent PT after a GTR of an SFT WHO grade 2 located in the left parietal lobe. Preoperatively, the patient presented with paresthesia and intermittent right-sided hemiparesis. Proton therapy was performed with 33 fractions to a total dose of 59.4 Gy (RBE). At the end of treatment, acute toxicities were limited to grade 1 dermatitis and grade 2 alopecia, with persistent mild hypesthesia in the right leg. Nine months post-PT, the patient developed progressive hypesthesia and weakness in the right lower extremity. Follow-up MRI showed no evidence of tumor recurrence and only nonspecific changes such as a small T2-weighted fluid-attenuated inversion recovery (FLAIR) hyperintensity in the treated area without contrast enhancement. Despite corticosteroid therapy and rehabilitation, the patient had stable symptoms with a grade 3 motor neuropathy. The second patient with a high-grade late toxicity had an intradural extramedullary SFT WHO grade II at the T3 spinal level and initially presented with paraparesis. Following GTR, the patient recovered completely from the initial symptoms and subsequently received adjuvant PT in 2 phases: 25 fractions to 50 Gy (RBE) covering the initial tumor bed with a 2 cm margin, followed by a boost to the high-risk region up to a total dose of 64 Gy (RBE). The spinal cord received a surface dose of 58.48 Gy (RBE) and a central dose of 53.95 Gy (RBE). The patient tolerated the PT well with low-grade acute toxicity (fatigue grade 1, dermatitis grade 1) and no new neurological symptoms. However, 2 months post-PT, the patient developed myelitis grade 1, which progressed to grade 4 myelitis by 6 months. Despite treatment with high-dose corticosteroids, hyperbaric oxygen therapy, and bevacizumab, the patient showed no clinical improvement and was left with persistent paraparesis, neuropathic pain, and neurogenic bladder dysfunction. This severe late toxicity was likely multifactorial, with potential contributions from prior spinal cord compression and ongoing nicotine use, both known risk factors for radiation-induced spinal cord injury.

**Table 3:** Acute and late toxicity comparing cranial and extracranial tumor locations.

Characteristic	Cranial (*n* = 11)	Extracranial (*n* = 6)
**Acute toxicity**		
** Prevalence, *n* (%)**	11 (100)	6 (100)
** Acute toxicity (type)**	Grade 1 - Dermatitis (*n* = 8) - Alopecia (*n* = 3) - Fatigue (*n* = 3) - Cognitive disturbance (*n* = 1) - Hyperpigmentation (*n* = 1) - Middle ear inflammation (*n* = 1) - Sinus disorder (*n* = 1) - Epistaxis (*n* = 1) Grade 2 - Alopecia (*n* = 3) - Dermatitis (*n* = 1)	Grade 1 - Fatigue (*n* = 5) - Dermatitis (*n* = 4) - Myelitis (*n* = 1) Grade 2 - Dermatitis (*n* = 1)
**Late toxicity**		
** Prevalence, *n* (%)**	5 (45)	2 (33)
** Late toxicity (type)**	Grade 1: - Alopecia (*n* = 1) - Osteonecrosis (*n* = 1) - Leukoencephalopathy (*n* = 1) Grade 2: - Sensory neuropathy (*n* = 1) - Sleep disorder (*n* = 1) - Cognitive disturbance (*n* = 2) - Hearing impairment (*n* = 1) - Hypopituitarism (*n* = 1) - Fatigue (*n* = 1) Grade 3: Motor neuropathy (*n* = 1)	Grade 1: - Alopecia (*n* = 1) - Hyperpigmentation (*n* = 1) Grade 4: Myelitis (*n* = 1)

### Quality of life

For 8 out of the 11 patients treated with a cranial SFT, QoL data were available. The prospectively collected questionnaires were evaluated until 3 years after PT. Of the 8 patients with available QoL datasets, 6 were treated in the primary adjuvant setting and the remaining 2 were treated at the time point of recurrence. Questionnaire compliance rates were 100% (*n* = 8) at baseline (T1), during PT (T2), and at the completion of treatment (T3). Longitudinal FU data were available for 50% (*n* = 4) at 1 year (T4) and 25% (*n* = 2) at both 2 years (T5) and 3 years (T6) post-treatment. Overall, the global health domain of the EORTC QLQ-C30 appeared broadly stable over time and showed no substantial difference compared to the general European population (Figure 3A). Similarly, the fatigue domain of the QLQ-C30 was stable over time and comparable to the general population (Figure 3B). In the domain of cognitive function, the mean values were constant over time and slightly lower than the values of the reference population (Figure 3C). The EORTC QLQ-BN20 hair loss score showed an increase during the period of PT, indicating acute toxicity, but returned to baseline levels 2 years post-treatment (Figure 3D).

**Figure 3:**
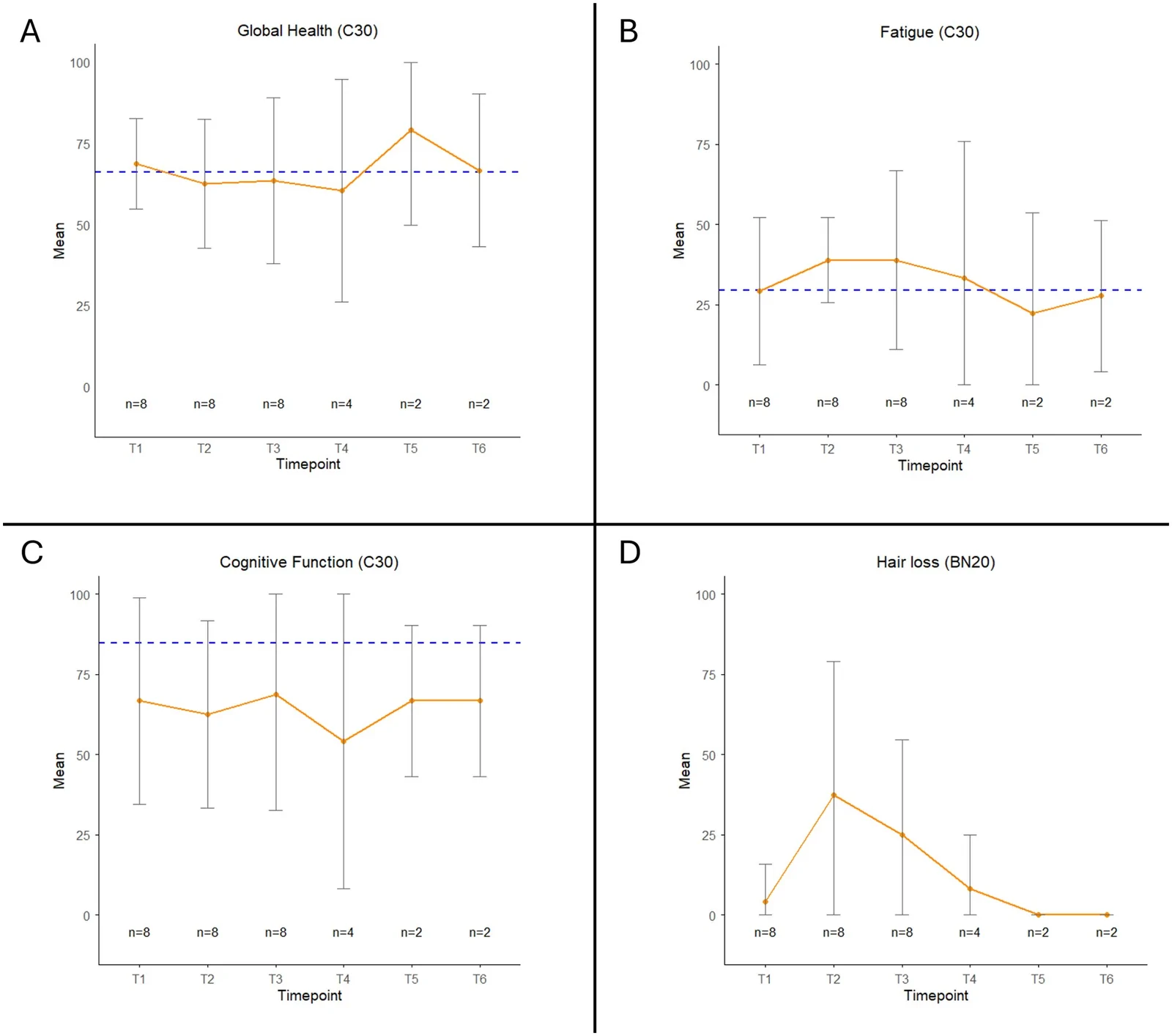
QoL assessed using the EORTC QLQ-C30 and BN20 questionnaires: global health status (A), fatigue (B), cognitive functioning (C), and hair loss (D) measured at baseline (T1), during PT (T2), at the end of treatment (T3), and annually up to 3 years post-treatment (T4-T6). The dotted line represents normative values for the general population, as reported by Nolte et al.^13^

## Discussion

This retrospective single-center study provides the largest analyzed cohort of patients with cranial and extracranial SFTs treated with PT to our knowledge. Previously, evidence for PT in SFT patients was based on case reports^14^^,^^15^ and studies including mixed cohorts treated with various RT modalities^5^^,^^16^^,^^17^ (Table 4). After a median FU of 47 months, we observed no deaths and an estimated 5-year LC rate of 81.8%. Proton therapy was well tolerated, with 2 (11%) patients presenting with severe late toxicity. Additionally, patient-reported QoL remained stable over time. These findings suggest that PT is feasible in selected patients with SFT, but the results should be interpreted as descriptive and hypothesis generating.

**Table 4:** Overview of published studies on SFTs that include patients treated with proton therapy.

References	Treatment period	Patients (total/PORT/PT)	Median FU (range)	OS	PFS	Toxicity
**Ton et al^5^**	2000-2020	34/34/10	60 months (0-282)	Not reported	1 y: 100% 3 y: 96% 5 y: 86%	Acute: grade 1-2 Late: 11% RICE grade II
**Melone et al^16^**	1980-2010	43/21/5	118 months (24-384)	1 y: 100% 5 y: 94% 10 y: 72%	1 y: 98% 5 y: 51% 10 y: 29%	Not reported
**Rutkowski et al^17^**	1989-2010	40/19/3	Not reported (2-408)	1 y: 100% 5 y: 92% 10 y: 68%	1 y: 96% 5 y: 49% 10 y: 28%	Not reported

Abbreviations: FU = Follow-up; OS = Overall survival; PFS = progression-free survival; PORT = postoperative radiotherapy; PT = proton therapy; RICE = radiation-induced contrast enhancement.

SFTs are rare mesenchymal neoplasms, accounting for <1% of all primary CNS tumors and are associated with variable clinical behavior. Surgical resection, particularly GTR, remains the cornerstone of therapy.^2^^,^^18–20^ There is evidence that SFT most probably recurs locally, especially following STR.^21^ However, the benefit of PORT has been debated. Several studies have demonstrated improved outcomes with PORT, particularly when combined with STR^21–24^ and even following GTR in high-risk patients.^19^^,^^25–29^ Conversely, other studies have found no significant survival advantage with PORT following STR^16^ or GTR.^2–4^ A recent systematic review by Na et al compared GTR alone with GTR + PORT in patients with intracranial SFT.^1^ The results showed a significant superiority of PORT over GTR alone in terms of OS and PFS. After 3-, 5-, and 10 years, the OS was 98.3%, 96.6%, and 81.4% in the GTR + PORT cohort and 93.2%, 88.5%, and 70.3% in the GTR-alone cohort, respectively. The benefit of PORT was consistent across grade 2 and grade 3 intracranial SFT. In our cohort, the observed OS was 100% over a median FU of 47 months. While these preliminary outcomes are encouraging, the small sample size and limited FU duration restrict the utility of our dataset strictly to descriptive statistics, precluding any formal comparative analysis against long-term benchmarks from larger systematic reviews.

When assessing the efficacy of PORT, LC is an important endpoint. In a 10-year retrospective study of intracranial SFTs, Lee et al^21^ reported significantly higher LC with PORT (63.4%) compared to surgery alone (27.9%, *P* = .0001). Similarly, Roohani et al^30^ reported 5- and 10-year LC rates following surgery and PORT of 80% in a small retrospective study with meningeal SFT. These stand in line with Ghia et al^27^ reported a 5-year LC rate of 92% in patients after GTR + PORT. In extracranial SFT, Krengli et al^31^ showed a significant association in 5-year LC rates after surgery + PORT vs surgery alone (88.0% vs 72.5%, *P* = .03). Only a few studies have addressed the role of PT in SFT. One small retrospective study including 5 patients treated with PT after STR showed an LF rate of 36.3% after GTR + PORT and 60% after STR + PORT.^16^ Ton et al^5^ reported outcomes of 29 patients with intracranial SFT including 11 patients treated with particle therapy in the primary setting (10 with protons and 1 with carbon ions). Overall, 5-year local PFS was 95.8%. Additionally, the authors reported a significant difference in LF, favoring particle therapy over photon RT (odds ratio (OR) = 0.038; 95% CI, 0.004-0.384; *P* = .002). However, patients treated with particle therapy received higher median doses (60 Gy vs 56.4 Gy), suggesting dose may have influenced outcomes. Indeed, higher radiation doses (≥60 Gy) were associated with improved in-field and out-of-field control (OR = 0.145; 95% CI, 0.029-0.742; *P* = .027). Across studies, recommended dose levels vary widely, ranging from >50 Gy up to ≥72 Gy.^22^^,^^27^ Our LC rates after a median FU of nearly 4 years are consistent with those previously reported. Although, we found no significant association of LC and total dose, WHO grade, tumor localization, and PT timing. Despite no significant difference, all 3 LFs occurred in patients with an STR, suggesting a potential advantage of GTR followed by adjuvant PT. Notably, 7 patients (41.2%) developed an LF after the initial resection without adjuvant treatment.

Given the predominantly local recurrence pattern of SFTs, it is important to assess the distribution of LF pattern, particularly in relation to the target volume coverage. In a recently published study by Ton et al, the overall recurrence rate was 48.3% after surgery and PORT.^5^ Recurrences were in-field in 42.9% and out-of-field in 57.1%. Notably, in the subgroup of patients treated with PT, no in-field failures were observed and only 1 patient developed an out-of-field recurrence. Such pattern of failure analysis is important to provide guidance for dose prescription and safety margins. This is further supported by evidence suggesting that the extent of target volume margins can directly influence LC. Lee et al^21^ demonstrated that smaller target volume margins (0-5 mm) were associated with significantly worse LC in comparison with larger margins (10-20 mm) (*P* = .021). In our cohort, an LF rate of 17.6% (*n* = 3) was observed following PT, presenting with 2 in-field recurrences and 1 out-of-field failure. Given the descriptive nature of our study and the small sample size, these rates cannot be directly compared against historical series.

In addition to efficacy, treatment-related toxicity is a crucial aspect when evaluating the clinical value of PT. Toxicity data for PORT in patients with SFT are limited. According to Ton et al, adjuvant RT was generally tolerated well with only grade I or II acute toxicities and no late adverse events. One patient developed a radiation-induced contrast enhancement post-PT, which was successfully treated with dexamethasone. Our findings confirmed the good tolerability of the treatment with only grade I and II acute side effects. However, 2 patients in our cohort experienced high-grade late toxicity. One patient developed grade 4 myelitis post-PT. Although generally accepted QUANTEC dose constraints were respected, the estimated risk of myelopathy at 60 Gy with conventional fractionated RT is around 6%.^32^ Furthermore, the patient had additional risk factors including a history of spinal cord compression and a persisting smoking status.^33^ To date, only case reports have described intradural extramedullary SFTs, and there is currently no standardized treatment approach for this rare entity.^34^ To our knowledge, this is the first reported case of an intradural extramedullary SFT treated with PT. The second patient with a severe late toxicity developed a grade 3 motor neuropathy. This occurred despite careful treatment planning, with no significant hotspots in normal brain tissue and no excessive dose to organs at risk. Overall, the estimated risk of symptomatic brain necrosis is about 5%.^35^ The occurrence of severe late complications in 11.8% of patients underscores that high-dose PT near the CNS axis carries an inherent risk of permanent neurological damage. While compounding factors such as mechanical compression and nicotine use were present in the myelitis case, these events demonstrate that PT cannot entirely eliminate normal tissue injury. This highlights the critical need not only for conservative OAR constraints and meticulous patient selection but also for a deeper understanding of particle radiobiology when escalating doses in these vulnerable regions. Notably, these high-grade toxicities occurred in patients who had not undergone prior re-irradiation, emphasizing that more robust data are required to inform clinical decision-making for high-risk anatomical sites. While OS and LC are key endpoints, patient-centered outcomes such as QoL are increasingly recognized as essential in the management of tumors. To date, few studies have prospectively assessed QoL in patients with this rare disease, making our findings a valuable contribution. Our descriptive findings suggested that QoL remained stable and aligned with reference population norms within the small subset of patients evaluated for cranial SFT. Regarding cognitive function, our results indicated stable but lower scores compared to the general population. These findings align with existing evidence in meningioma patients, where long-term neurocognitive impairment has been documented.^36^^,^^37^ This is likely an effect of surgery, which does not appear to be further exacerbated by additional RT. To our knowledge, only one other study evaluated QoL in intracranial SFT treated with surgery alone as primary therapy. Yu et al^29^ demonstrated a decline in QoL among patients with tumor progression and metastasis. However, meaningful comparisons across studies remain challenging because of differences in QoL assessment tools, heterogeneous patient cohorts, and the limited number of patients and events.

Several limitations should be considered when interpreting these findings. First, this was a retrospective single-center analysis with a small sample size, reflecting the rarity of SFT and the limited number of patients treated with PT. Consequently, OS and LC estimates are descriptive, and subgroup analyses are markedly underpowered, particularly given the low number of LF events. Second, the cohort was clinically heterogeneous, including intracranial and extracranial tumor sites, primary postoperative and recurrent treatment settings, variable extents of resection, 2 patients treated in a salvage re-irradiation setting, and 2 patients receiving concomitant systemic therapy. These factors may confound interpretation of oncological outcomes, toxicity, and QoL. Third, the long inclusion period from 2002 to 2024 spans changes in diagnostic terminology, WHO classification, treatment techniques, and FU practices. Comprehensive centralized histopathological and molecular reclassification according to contemporary standards was not possible because of limited archival tissue availability, and STAT6 status and WHO grade were unavailable in a subset of patients. Fourth, most patients underwent FU at external referring institutions. Although imaging and documented toxicities were re-reviewed internally, heterogeneity in imaging protocols and external documentation may have affected response assessment and may have led to underreporting of low-grade late toxicities. Finally, QoL analyses were limited to a small subset of patients with cranial SFT, and questionnaire availability decreased substantially during long-term FU. Therefore, QoL findings should be interpreted as exploratory and descriptive only. In addition, the absence of a photon-treated comparator group precludes any direct conclusions regarding clinical superiority or normal tissue-sparing benefits of PT. Overall, these results should be regarded as hypothesis generating and require validation in larger multicenter or registry-based cohorts.

Future studies should focus on comparison of proton and photon therapy and on determining the optimal timing of RT. Nearly half of our patients had multiple resections prior to PT, raising the question of whether early PORT could prevent recurrence by targeting microscopic disease. Moreover, integrating molecular profiling and imaging biomarkers may enhance risk stratification. Due to the rarity of SFTs, international registries or collaborative studies are essential to enable more robust outcome analyses and refine treatment strategies.

## Conclusion

In this retrospective single-center cohort, postoperative pencil beam scanning PT was associated with encouraging preliminary LC in selected patients with cranial and extracranial SFTs. Acute toxicity was limited to grade 1-2 events; however, severe late neurological toxicity occurred in 2 patients, highlighting the need for careful patient selection and conservative OAR constraints, particularly for tumors adjacent to the CNS axis. QoL outcomes in the cranial subgroup appeared broadly stable among patients with available questionnaires, but these findings should be interpreted descriptively because of the small sample size and decreasing questionnaire availability over time.

## Supplementary Material

tzag023_Supplementary_Data
