# Supplementary material for: Clinical outcomes and quality of life after proton therapy for cranial and extracranial solitary fibrous tumors
Source: BJR Open. 2026 Oct 26;8(1):tzag023. doi: 10.1093/bjro/tzag023 (PMC13730268; doi:10.1093/bjro/tzag023)
Supplement: Supplementary file 1 [file tzag023_supplementary_data.zip › Supplementary_Data/Appendices_R3_9.docx]

**Appendix A: Short descriptions of the other three pollution-caused diseases**

In 1965, residents living along the Agano River in Niigata Prefecture reported symptoms similar to Minamata disease (referred to as Niigata Minamata disease) due to the consumption of fish and shellfish from the river contaminated with methylmercury discharged from the plant of Showa Denko K.K. A total of 716 patients had been officially recognized as victims of Niigata Minamata disease (Niigata Prefecture 2024).

Itai-itai disease represents another significant illness resulting from pollution: untreated wastewater from the plant operated by Mitsui Mining and Smelting led to cadmium contamination for residents living downstream of the Jinzu River in Toyama Prefecture. The initial case was identified in 1955. The Ministry of Health and Welfare officially established the causal link between cadmium pollution and the disease in 1968 and acknowledged 96 victims by 1970 (Environment Agency 1973).

In Yokkaichi, a prominent industrial development region in Mie Prefecture, residents suffered from severe asthma due to the uncontrolled sulfur dioxide emissions from the clustered facilities of chemical, oil, and power generation companies, including Mitsubishi Petrochemical Corporation and Chubu Electric Power Co., Inc. (Prime Minister’s Office 1971). The local government, which had invited these firms to promote regional development, introduced Japan’s first municipal medical-aid scheme for pollution victims in 1965.

**Appendix B: Relative change of the proportions of directors from the 1960**–**1969 cohort and firms’ symbolic adaptation (2003 = 1)**

Notes: Both series are indexed to 1 in 2003. Markers indicate annual observed values, and lines represent LOWESS-smoothed trends.

**Appendix C: Predicted probabilities of symbolic adaptation and tests of non-executive directors as a moderator (Hypothesis 2)**

**Appendix D: Predicted probabilities of symbolic adaptation and tests of activist shareholders as a moderator (Hypothesis 3)**

**Appendix E: Robustness checks**

We conduct the following robustness checks to assess the validity and reliability of the results in Tables 2 and 3.

**E1: Fine-grained periodization**

We refine the cohort definitions to assess the sensitivity of our results and capture more nuanced effects of director-cohort imprinting on symbolic adaptation. We use nine cohort categories rather than four, create a series of variables that count the number of directors from each cohort, and estimate models using these variables. The results are reported in Appendix E Tables 4 and 5. The dependent variables used in Appendix E Tables 4 and 5 are residualized measures constructed by accounting for industry and firm fixed effects, respectively. We use a 0.85 S.D. threshold in creating the dependent variables. In Models 12 to 15, we find that the coefficients for *Cohort 1964–1967* are positive and significant (*p* < 0.05 or better), suggesting that directors from this specific cohort within the 1960s were particularly imprinted with symbolic approaches. Although the levels of significance decrease in Model 13, in which we test interaction effects (*p* < 0.10), the directions of the effects are consistent with those reported in Tables 2 and 3. In Appendix E Table 5, most cohort coefficients are not statistically significant; however, the signs of the focal variables align with our theoretical expectations.

Furthermore, in Appendix E Tables 6 and 7, we adopt an alternative cohort categorization and create two cohorts for directors who entered the corporate world in the 1960s: *Cohort 1960–1964* and *Cohort 1965–1969*. In these tables, we also estimate residualized measures constructed from models with industry and firm fixed effects, respectively. We use the 0.85 S.D. threshold in constructing the dependent variables. The results are broadly consistent with those reported in Appendix E Tables 4 and 5. In Appendix E Table 6, the coefficients for *Cohort 1965–1969* are positive and significant (*p* < 0.05 or better), and the interaction terms in Models 23 and 25 are negative and significant (*p* < 0.05). Although the significance of the interaction effect weakens in Models 28 and 30 (*p* < 0.10) in Appendix E Table 7, the direction of the effect remains consistent with our main findings.

Although the moderating effects of *activist shareholder* do not receive significant support in these robustness checks, the overall patterns are broadly consistent with our main findings. The results for the later periods of the 1960s may reflect the fact that these directors began their careers amid heightened public and social scrutiny of pollution, culminating in the 1970 ‘Pollution Diet’. Firms facing intensified criticism during this period were especially prone to symbolic adaptation; directors who entered then were more likely to be exposed to such responses.

**E2: Interaction effects of NEDs**

Our main analyses show that the effects associated with the 1960–1969 cohort are attenuated when firms are subject to oversight by non-executive directors (NEDs) (Hypothesis 2). Building on this result, we examine heterogeneity among NEDs by distinguishing those who were exposed to similar social and historical conditions during the 1960s, which constituted their formative periods. To do so, we create a measure that counts the number of NEDs who were 22 years old during the 1960s (i.e. *Cohort 1960–1969 NED*) and estimate its interaction with our primary independent variable (*Cohort 1960–1969*). Because many NEDs did not begin their careers in the corporate sector and instead entered professions such as law, accounting, academia, or public administration, we identify entry into professional careers—rather than corporate employment—as the relevant sensitive period for imprint formation.

We present the results in Appendix E Tables 8 and 9. The dependent variables used in Appendix E Tables 8 and 9 are residualized measures constructed by accounting for industry and firm fixed effects, respectively. These analyses yield two key findings. First, the coefficients for cohort-matched non-executive directors (*Cohort 1960–1969 NED*) are consistently insignificant across all model specifications. Moreover, including this variable does not materially alter the estimated effects of our focal independent variable (*Cohort 1960–1969*) or the interaction term between *Cohort 1960–1969* and *Activist shareholder*.

Second, the interaction between *Cohort 1960–1969* and *Cohort 1960–1969 NED* is also insignificant across models. We conducted additional unreported analyses using a binary indicator of cohort-matched NED imprinting and obtained similarly insignificant results.

Taken together, we find no statistically significant moderating effect of cohort-matched NEDs from the 1960s. In contrast, as documented in the main analyses, we consistently observe a robust and statistically significant negative interaction between director cohort (1960–1969) and the presence of NEDs across all models including the NED interaction (*Cohort 1960*–*1969 × NED*) in Tables 2 and 3. This pattern suggests that NED representation systematically conditions the extent to which director-cohort imprints shape firms’ propensity to engage in symbolic adaptation.

We offer three interpretations for the insignificant interaction effects involving the 1960s cohort of NEDs. First, because NEDs primarily serve as monitors and independent advisors to corporate leadership, their influence may be relatively insensitive to generational cohort differences. Second, most NEDs originate from professional rather than corporate career paths and therefore experience different entry timings and formative periods. Consequently, our assumption that imprint formation occurs uniformly at age 22 may oversimplify the imprinting process for this group. Third, professional careers are often governed by strong occupational norms that differ from those prevalent in corporate internal labor markets, which historically emphasized economic growth and performance. These professional norms, whether they encourage or discourage symbolic forms of environmental adaptation, remain consistent across time periods.

**E3: CEO imprinting effects**

Since CEOs might be more influential than other board members, we examined CEO imprinting effects by constructing a dummy variable indicating whether a firm’s CEO belongs to the 1960s cohort. We report the corresponding results in Appendix E Tables 10 and 11. These tables report estimates based on residuals computed with industry fixed effects (Appendix E Table 10) and firm fixed effects (Appendix E Table 11). Focusing on the coefficients relevant to CEO imprinting, we find positive and marginally significant effects in Models 41, 42, and 44 of Appendix E Table 10 (*p* < 0.10). Although the corresponding coefficients are insignificant in Appendix E Table 11, they are consistently positive across both tables, providing directional support for our theoretical predictions.

We interpret the weaker CEO imprinting effects, relative to those of the 1960s director cohort, as reflecting the limited decision-making authority typically exercised by CEOs in Japanese firms. As Olcott (2009) notes, “a Japanese CEO is not a primary decision-maker or agent of change, but rather a senior figure who facilitates consensus and encourages bottom-up decision making” (pp. 229–230). Consistent with this view, Olcott (2009) highlights the influential role of boards in Japanese corporations, emphasizing that boards engage actively in strategic deliberation and are less likely to serve as symbolic rubber stamps.

Accordingly, generational imprints embedded in the collective makeup of boards are more likely to be translated into managerial decisions than the imprints of individual CEOs. This pattern may align with the consensus-oriented decision-making processes that typify Japanese corporate governance.

We also find that the interaction between CEO imprinting and activist shareholders is positive and significant in Models 43 and 44 of Appendix E Table 10. This pattern differs from the negative moderating effect observed in our main analysis. This difference may reflect the distinct roles of individual CEO imprinting and collective board-level imprinting in Japanese firms.

**E4: Using alternative estimation specifications: Two-way fixed-effects logistic regressions**

We also estimate two-way fixed-effects logistic regression models and report the results in Appendix E Tables 12 and 13. The dependent variables used in Appendix E Tables 12 and 13 are residualized measures constructed by accounting for industry and firm fixed effects, respectively. The results are largely consistent with our main findings. In Appendix E Table 12, the number of directors from cohorts imprinted during the era of ceremonial compliance is significantly associated with symbolic environmental management (*p* < 0.001). Moreover, this relationship is weaker when boards have greater representation of non-executive directors (*p* < 0.01) and when activist shareholders hold larger ownership stakes (*p* < 0.05). The estimations in Appendix E Table 13 yield a similar pattern. Although the moderating effect of activist shareholder ownership in Model 55 is significant only at the 0.10 level and becomes insignificant in Model 56, its direction remains consistent with our theoretical prediction. Other findings in Models 53 and 54 remain robust. These analyses therefore provide additional evidence that our substantive conclusions are not sensitive to an estimation approach designed specifically for binary outcomes.

**E5: Continuous measures of the dependent variables**

In addition to the dichotomous measures of symbolic adaptation, we reestimate our models using a continuous dependent variable. We construct this measure by recoding all positive residuals to zero and then multiplying the remaining values by –1, such that higher values correspond to greater levels of symbolic adaptation. We report the results in Appendix E Tables 14 and 15. Across all specifications, we consistently find a significant positive effect of our main independent variable (*Cohort 1960–1969*). The interaction with NEDs (*Cohort 1960–1969 × NED*) is negative and marginally significant (*p* < 0.10), and the interaction with activist shareholders (*Cohort 1960–1969 × Activist shareholder*) is negative and significant (*p* < 0.01). These results align closely with our theoretical expectations and indicate that our findings are robust across alternative operationalizations of the dependent variable.

**E6: Alternative cutoffs in creating the dependent variables**

In constructing the residual-based dependent variables used in Tables 2 and 3, we apply the 0.85 S.D. threshold. To further assess the sensitivity and validity of our results to this threshold choice, we reestimate all models using 0.70 S.D. and 1.00 S.D. thresholds in Appendix E Tables 16 and 17. Appendix E Table 16 reports models in which, for each year, we predict Nikkei Environmental Performance using industry fixed effects and apply the 0.70 S.D. (Models 65–68) and 1.00 S.D. (Models 69–72) thresholds to identify firms engaging in symbolic approaches. These results are comparable to those reported in Tables 2 and 3. The coefficients of *Cohort 1960–1969* are positive and significant (*p* < 0.05 or better), and both interaction terms are negative and significant (*p* < 0.10 or better). In Appendix E Table 17, we pool observations across all years and use firm fixed effects when predicting Nikkei Environmental Performance. Support for our predictions is weaker in Models 73–76 with the 0.70 S.D. threshold. However, Models 77–80 using the 1.00 S.D. threshold provide support for Hypothesis 1 (*p* < 0.05 or better) and show two negative and significant interaction terms (*p* < 0.10 or better). Overall, these analyses indicate that our principal results are robust to alternative threshold specifications.

**Appendix E Table 4: Results of two-way fixed-effects regressions with D.V. estimated from models with industry fixed effects, using four-year imprinting windows**

| D.V. | S.D. = 0.85, using industry fixed effects | | | | |
| --- | --- | --- | --- | --- | --- |
| Variables | Model 11 | Model 12 | Model 13 | Model 14 | Model 15 |
| Lambda | -2.442 | -2.760+ | -2.742+ | -2.759+ | -2.738+ |
|  | [1.516] | [1.485] | [1.494] | [1.483] | [1.492] |
| Unabsorbed slack | 0.005 | 0.005 | 0.009 | 0.005 | 0.010 |
|  | [0.014] | [0.015] | [0.016] | [0.015] | [0.016] |
| HR slack | -1.758 | -1.820 | -1.842 | -1.745 | -1.732 |
|  | [2.721] | [2.723] | [2.735] | [2.770] | [2.789] |
| Absorbed slack | 0.001 | 0.003 | -0.002 | 0.003 | -0.003 |
|  | [0.161] | [0.161] | [0.161] | [0.161] | [0.162] |
| Performance | -0.002** | -0.002** | -0.002** | -0.002** | -0.002** |
|  | [0.001] | [0.001] | [0.001] | [0.001] | [0.001] |
| Log of firm age | 0.283* | 0.262* | 0.266* | 0.263* | 0.269* |
|  | [0.103] | [0.099] | [0.097] | [0.099] | [0.097] |
| Log of firm size | -0.218+ | -0.238+ | -0.243+ | -0.237+ | -0.241+ |
|  | [0.116] | [0.114] | [0.114] | [0.114] | [0.114] |
| Tenure | 0.005 | 0.004 | 0.004 | 0.004 | 0.004 |
|  | [0.003] | [0.003] | [0.003] | [0.003] | [0.003] |
| CEO tenure | 0.002 | 0.002 | 0.002 | 0.001 | 0.002 |
|  | [0.002] | [0.002] | [0.002] | [0.002] | [0.002] |
| CEO tenure missing | -0.020 | -0.016 | -0.017 | -0.016 | -0.016 |
|  | [0.036] | [0.036] | [0.036] | [0.036] | [0.036] |
| Political background | -0.027 | -0.027 | -0.028 | -0.028 | -0.030 |
|  | [0.027] | [0.027] | [0.028] | [0.027] | [0.028] |
| Production background | 0.003 | 0.000 | 0.000 | 0.000 | 0.000 |
|  | [0.009] | [0.008] | [0.008] | [0.007] | [0.007] |
| Cohort 1964–1967 NED | 0.000 | -0.018 | 0.002 | -0.018 | 0.004 |
|  | [0.013] | [0.013] | [0.015] | [0.013] | [0.016] |
| NED | -0.026* | -0.025* | -0.015 | -0.025* | -0.015 |
|  | [0.011] | [0.011] | [0.011] | [0.011] | [0.011] |
| Activist shareholder | -0.028 | -0.023 | -0.024 | -0.094 | -0.130 |
|  | [0.067] | [0.066] | [0.067] | [0.131] | [0.129] |
| Cohort –1955 |  | 0.019 | 0.021 | 0.019 | 0.021 |
|  |  | [0.016] | [0.016] | [0.016] | [0.016] |
| Cohort 1956–1959 |  | -0.015 | -0.013 | -0.015 | -0.013 |
|  |  | [0.015] | [0.016] | [0.015] | [0.015] |
| Cohort 1960–1963 |  | -0.006 | -0.007 | -0.005 | -0.006 |
|  |  | [0.010] | [0.010] | [0.010] | [0.010] |
| Cohort 1964–1967 |  | 0.014* | 0.021** | 0.014* | 0.020** |
|  |  | [0.005] | [0.006] | [0.005] | [0.006] |
| Cohort 1968–1971 |  | 0.003 | 0.003 | 0.004 | 0.004 |
|  |  | [0.004] | [0.004] | [0.003] | [0.003] |
| Cohort 1972–1975 |  | 0.000 | -0.001 | 0.000 | -0.001 |
|  |  | [0.005] | [0.005] | [0.005] | [0.005] |
| Cohort 1976–1979 |  | -0.004 | -0.004 | -0.004 | -0.004 |
|  |  | [0.005] | [0.005] | [0.005] | [0.005] |
| Cohort 1980–1983 |  | 0.003 | 0.003 | 0.003 | 0.002 |
|  |  | [0.006] | [0.006] | [0.006] | [0.006] |
| Cohort 1984– |  | -0.021+ | -0.022+ | -0.021 | -0.022+ |
|  |  | [0.012] | [0.012] | [0.012] | [0.012] |
| Cohort 1964–1967 |  |  | -0.010+ |  | -0.011+ |
| × NED |  |  | [0.005] |  | [0.006] |
| Cohort 1964–1967 |  |  |  | 0.028 | 0.042 |
| × Activist shareholder |  |  |  | [0.039] | [0.037] |
| Constant | 3.133 | 3.661 | 3.669 | 3.642 | 3.642 |
|  | [2.405] | [2.343] | [2.346] | [2.349] | [2.351] |
| Firm FE | Yes | Yes | Yes | Yes | Yes |
| Year FE | Yes | Yes | Yes | Yes | Yes |
| N | 4822 | 4822 | 4822 | 4822 | 4822 |
| R-squared | 0.42 | 0.43 | 0.43 | 0.43 | 0.43 |
| Log Likelihood | -930.06 | -919.84 | -915.14 | -919.19 | -913.75 |

Notes: Standard errors two-way clustered by firm and year are reported in brackets.

+ p<0.10, * p<0.05, ** p<0.01, *** p<0.001. In constructing the dependent variables, we use residuals obtained from year-specific regression models that include one-digit Nikkei industry codes.

**Appendix E Table 5: Results of two**-**way fixed**-**effects regressions with D.V. estimated from models with firm fixed effects, using four**-**year imprinting windows**

| D.V. | S.D. = 0.85, using firm fixed effects | | | | |
| --- | --- | --- | --- | --- | --- |
| Variables | Model 16 | Model 17 | Model 18 | Model 19 | Model 20 |
| Lambda | -1.955 | -1.958 | -1.936 | -1.957 | -1.934 |
|  | [1.795] | [1.807] | [1.803] | [1.806] | [1.801] |
| Unabsorbed slack | 0.001 | 0.001 | 0.005 | 0.001 | 0.005 |
|  | [0.022] | [0.022] | [0.022] | [0.022] | [0.022] |
| HR slack | -1.129 | -1.226 | -1.252 | -1.195 | -1.180 |
|  | [3.302] | [3.353] | [3.371] | [3.367] | [3.393] |
| Absorbed slack | 0.116 | 0.126 | 0.119 | 0.126 | 0.119 |
|  | [0.185] | [0.182] | [0.182] | [0.182] | [0.182] |
| Performance | 0.000 | 0.000 | 0.000 | 0.000 | 0.000 |
|  | [0.001] | [0.001] | [0.001] | [0.001] | [0.001] |
| Log of firm age | 0.146 | 0.144 | 0.150 | 0.144 | 0.151 |
|  | [0.176] | [0.177] | [0.175] | [0.177] | [0.175] |
| Log of firm size | -0.192 | -0.191 | -0.197 | -0.191 | -0.196 |
|  | [0.156] | [0.157] | [0.156] | [0.157] | [0.156] |
| Tenure | 0.000 | 0.001 | 0.001 | 0.001 | 0.001 |
|  | [0.005] | [0.005] | [0.005] | [0.005] | [0.005] |
| CEO tenure | 0.001 | 0.000 | 0.000 | 0.000 | 0.000 |
|  | [0.002] | [0.002] | [0.002] | [0.002] | [0.002] |
| CEO tenure missing | 0.025 | 0.026 | 0.025 | 0.026 | 0.025 |
|  | [0.048] | [0.048] | [0.048] | [0.048] | [0.048] |
| Political background | 0.017 | 0.012 | 0.011 | 0.012 | 0.010 |
|  | [0.039] | [0.039] | [0.039] | [0.038] | [0.039] |
| Production background | -0.008 | -0.009 | -0.009 | -0.009 | -0.010 |
|  | [0.009] | [0.009] | [0.009] | [0.009] | [0.009] |
| Cohort 1964–1967 NED | -0.002 | -0.014 | 0.010 | -0.014 | 0.011 |
|  | [0.018] | [0.020] | [0.023] | [0.020] | [0.023] |
| NED | -0.009 | -0.006 | 0.005 | -0.006 | 0.006 |
|  | [0.014] | [0.013] | [0.013] | [0.013] | [0.013] |
| Activist shareholder | 0.013 | 0.018 | 0.017 | -0.012 | -0.053 |
|  | [0.057] | [0.054] | [0.056] | [0.118] | [0.119] |
| Cohort –1955 |  | -0.004 | -0.002 | -0.004 | -0.001 |
|  |  | [0.030] | [0.031] | [0.030] | [0.031] |
| Cohort 1956–1959 |  | -0.042+ | -0.040+ | -0.042+ | -0.040+ |
|  |  | [0.023] | [0.023] | [0.023] | [0.023] |
| Cohort 1960–1963 |  | -0.005 | -0.006 | -0.005 | -0.006 |
|  |  | [0.013] | [0.013] | [0.013] | [0.013] |
| Cohort 1964–1967 |  | 0.007 | 0.015 | 0.007 | 0.015 |
|  |  | [0.008] | [0.008] | [0.008] | [0.008] |
| Cohort 1968–1971 |  | 0.001 | 0.001 | 0.001 | 0.001 |
|  |  | [0.005] | [0.005] | [0.005] | [0.005] |
| Cohort 1972–1975 |  | -0.003 | -0.003 | -0.003 | -0.003 |
|  |  | [0.006] | [0.006] | [0.006] | [0.006] |
| Cohort 1976–1979 |  | -0.005 | -0.005 | -0.005 | -0.005 |
|  |  | [0.006] | [0.006] | [0.006] | [0.006] |
| Cohort 1980–1983 |  | 0.011 | 0.010 | 0.011 | 0.010 |
|  |  | [0.009] | [0.009] | [0.009] | [0.009] |
| Cohort 1984– |  | -0.015 | -0.016 | -0.014 | -0.015 |
|  |  | [0.013] | [0.012] | [0.013] | [0.013] |
| Cohort 1964–1967 |  |  | -0.012* |  | -0.012* |
| × NED |  |  | [0.005] |  | [0.006] |
| Cohort 1964–1967 |  |  |  | 0.012 | 0.028 |
| × Activist shareholder |  |  |  | [0.035] | [0.036] |
| Constant | 3.075 | 3.082 | 3.093 | 3.074 | 3.075 |
|  | [3.106] | [3.138] | [3.121] | [3.145] | [3.126] |
| Firm FE | Yes | Yes | Yes | Yes | Yes |
| Year FE | Yes | Yes | Yes | Yes | Yes |
| N | 4822 | 4822 | 4822 | 4822 | 4822 |
| R-squared | 0.13 | 0.13 | 0.13 | 0.13 | 0.13 |
| Log Likelihood | -1897.8 | -1891.23 | -1886.66 | -1891.16 | -1886.25 |

Notes: Standard errors two-way clustered by firm and year are reported in brackets.

+ p<0.10, * p<0.05, ** p<0.01, *** p<0.001. In constructing the dependent variables, we use residuals obtained from a regression model that pools observations across all years and includes firm fixed effects.

**Appendix E Table 6: Results of two-way fixed-effects regressions with D.V. estimated from models with industry fixed effects, using the early vs late 1960s imprinting windows**

| D.V. | S.D. = 0.85, using industry fixed effects | | | | |
| --- | --- | --- | --- | --- | --- |
| Variables | Model 21 | Model 22 | Model 23 | Model 24 | Model 25 |
| Lambda | -2.125+ | -2.620* | -2.641* | -2.608* | -2.626* |
|  | [1.191] | [1.188] | [1.195] | [1.190] | [1.197] |
| Unabsorbed slack | 0.003 | 0.002 | 0.005 | 0.002 | 0.005 |
|  | [0.014] | [0.014] | [0.014] | [0.014] | [0.014] |
| HR slack | -1.859 | -2.006 | -1.986 | -1.900 | -1.859 |
|  | [2.343] | [2.349] | [2.340] | [2.398] | [2.388] |
| Absorbed slack | 0.012 | -0.005 | -0.015 | -0.007 | -0.017 |
|  | [0.145] | [0.144] | [0.143] | [0.145] | [0.144] |
| Performance | -0.002** | -0.002** | -0.002** | -0.002** | -0.002** |
|  | [0.001] | [0.001] | [0.001] | [0.001] | [0.001] |
| Log of firm age | 0.254* | 0.217+ | 0.212+ | 0.218+ | 0.213+ |
|  | [0.112] | [0.116] | [0.114] | [0.116] | [0.113] |
| Log of firm size | -0.166 | -0.204* | -0.204* | -0.202* | -0.203* |
|  | [0.097] | [0.094] | [0.094] | [0.095] | [0.094] |
| Tenure | 0.005+ | 0.005 | 0.005 | 0.005 | 0.005 |
|  | [0.003] | [0.003] | [0.003] | [0.003] | [0.003] |
| CEO tenure | 0.001 | 0.001 | 0.001 | 0.001 | 0.001 |
|  | [0.002] | [0.002] | [0.002] | [0.002] | [0.002] |
| CEO tenure missing | -0.013 | -0.006 | -0.006 | -0.007 | -0.007 |
|  | [0.029] | [0.030] | [0.030] | [0.030] | [0.031] |
| Political background | -0.026 | -0.022 | -0.023 | -0.023 | -0.024 |
|  | [0.026] | [0.027] | [0.027] | [0.027] | [0.027] |
| Production background | 0.010 | 0.004 | 0.003 | 0.004 | 0.003 |
|  | [0.009] | [0.009] | [0.009] | [0.009] | [0.009] |
| Cohort 1965–1969 NED | 0.004 | -0.008 | 0.008 | -0.007 | 0.009 |
|  | [0.016] | [0.016] | [0.016] | [0.016] | [0.016] |
| NED | -0.026* | -0.026* | -0.011 | -0.027* | -0.011 |
|  | [0.010] | [0.010] | [0.010] | [0.010] | [0.010] |
| Activist shareholder | -0.058 | -0.057 | -0.057 | -0.149 | -0.166 |
|  | [0.067] | [0.067] | [0.067] | [0.118] | [0.114] |
| Cohort –1959 |  | 0.001 | 0.007 | 0.001 | 0.008 |
|  |  | [0.014] | [0.014] | [0.014] | [0.014] |
| Cohort 1960–1964 |  | 0.001 | -0.001 | 0.001 | 0.000 |
|  |  | [0.007] | [0.007] | [0.007] | [0.007] |
| Cohort 1965–1969 |  | 0.013* | 0.018** | 0.013* | 0.018** |
|  |  | [0.005] | [0.005] | [0.005] | [0.005] |
| Cohort 1970–1979 |  | 0.002 | 0.001 | 0.002 | 0.001 |
|  |  | [0.002] | [0.002] | [0.002] | [0.002] |
| Cohort 1980– |  | 0.003 | 0.002 | 0.003 | 0.002 |
|  |  | [0.006] | [0.006] | [0.006] | [0.005] |
| Cohort 1965–1969 |  |  | -0.008* |  | -0.008* |
| × NED |  |  | [0.003] |  | [0.003] |
| Cohort 1965–1969 |  |  |  | 0.038 | 0.046 |
| × Activist shareholder |  |  |  | [0.046] | [0.044] |
| Constant | 2.433 | 3.323+ | 3.352+ | 3.292 | 3.316 |
|  | [1.918] | [1.889] | [1.897] | [1.897] | [1.905] |
| Firm FE | Yes | Yes | Yes | Yes | Yes |
| Year FE | Yes | Yes | Yes | Yes | Yes |
| N | 5129 | 5129 | 5129 | 5129 | 5129 |
| R-squared | 0.41 | 0.41 | 0.41 | 0.41 | 0.41 |
| Log Likelihood | -1087.89 | -1081.20 | -1074.53 | -1080.22 | -1073.13 |

Notes: Standard errors two-way clustered by firm and year are reported in brackets.

+ p<0.10, * p<0.05, ** p<0.01, *** p<0.001. In constructing the dependent variables, we use residuals obtained from year-specific regression models that include one-digit Nikkei industry codes.

**Appendix E Table 7: Results of two-way fixed-effects regressions with D.V. estimated from models with firm fixed effects, using the early vs late 1960s imprinting windows**

| D.V. | S.D. = 0.85, using firm fixed effects | | | | |
| --- | --- | --- | --- | --- | --- |
| Variables | Model 26 | Model 27 | Model 28 | Model 29 | Model 30 |
| Lambda | -1.048 | -1.521 | -1.541 | -1.516 | -1.533 |
|  | [1.684] | [1.643] | [1.631] | [1.647] | [1.633] |
| Unabsorbed slack | 0.005 | 0.004 | 0.007 | 0.004 | 0.007 |
|  | [0.021] | [0.021] | [0.021] | [0.021] | [0.021] |
| HR slack | -0.210 | -0.557 | -0.537 | -0.511 | -0.472 |
|  | [3.163] | [3.168] | [3.133] | [3.211] | [3.173] |
| Absorbed slack | 0.086 | 0.081 | 0.072 | 0.081 | 0.071 |
|  | [0.173] | [0.175] | [0.172] | [0.176] | [0.173] |
| Performance | 0.000 | 0.000 | 0.000 | 0.000 | 0.000 |
|  | [0.001] | [0.001] | [0.001] | [0.001] | [0.001] |
| Log of firm age | 0.173 | 0.140 | 0.134 | 0.140 | 0.135 |
|  | [0.174] | [0.177] | [0.175] | [0.177] | [0.175] |
| Log of firm size | -0.136 | -0.173 | -0.174 | -0.172 | -0.173 |
|  | [0.139] | [0.137] | [0.137] | [0.138] | [0.138] |
| Tenure | 0.001 | 0.002 | 0.002 | 0.002 | 0.002 |
|  | [0.005] | [0.005] | [0.005] | [0.005] | [0.005] |
| CEO tenure | 0.000 | 0.000 | 0.000 | 0.000 | 0.000 |
|  | [0.002] | [0.002] | [0.002] | [0.002] | [0.002] |
| CEO tenure missing | 0.014 | 0.021 | 0.021 | 0.020 | 0.020 |
|  | [0.042] | [0.041] | [0.042] | [0.041] | [0.042] |
| Political background | 0.018 | 0.019 | 0.017 | 0.018 | 0.017 |
|  | [0.038] | [0.038] | [0.039] | [0.038] | [0.039] |
| Production background | 0.001 | -0.002 | -0.002 | -0.002 | -0.002 |
|  | [0.011] | [0.010] | [0.010] | [0.010] | [0.010] |
| Cohort 1965–1969 NED | 0.004 | -0.006 | 0.010 | -0.005 | 0.010 |
|  | [0.019] | [0.020] | [0.023] | [0.021] | [0.023] |
| NED | -0.010 | -0.009 | 0.005 | -0.009 | 0.005 |
|  | [0.014] | [0.014] | [0.013] | [0.014] | [0.013] |
| Activist shareholder | -0.006 | -0.005 | -0.004 | -0.044 | -0.061 |
|  | [0.057] | [0.056] | [0.056] | [0.100] | [0.098] |
| Cohort –1959 |  | -0.020 | -0.013 | -0.020 | -0.013 |
|  |  | [0.016] | [0.015] | [0.016] | [0.015] |
| Cohort 1960–1964 |  | 0.010 | 0.009 | 0.010 | 0.009 |
|  |  | [0.007] | [0.008] | [0.007] | [0.007] |
| Cohort 1965–1969 |  | 0.010 | 0.014* | 0.009 | 0.014* |
|  |  | [0.006] | [0.006] | [0.006] | [0.006] |
| Cohort 1970–1979 |  | -0.001 | -0.002 | -0.001 | -0.002 |
|  |  | [0.003] | [0.003] | [0.003] | [0.003] |
| Cohort 1980– |  | 0.004 | 0.003 | 0.004 | 0.003 |
|  |  | [0.008] | [0.008] | [0.008] | [0.008] |
| Cohort 1965–1969 |  |  | -0.008+ |  | -0.008+ |
| × NED |  |  | [0.004] |  | [0.004] |
| Cohort 1965–1969 |  |  |  | 0.016 | 0.024 |
| × Activist shareholder |  |  |  | [0.042] | [0.042] |
| Constant | 1.707 | 2.565 | 2.594 | 2.552 | 2.575 |
|  | [2.858] | [2.803] | [2.788] | [2.813] | [2.796] |
| Firm FE | Yes | Yes | Yes | Yes | Yes |
| Year FE | Yes | Yes | Yes | Yes | Yes |
| N | 5129 | 5129 | 5129 | 5129 | 5129 |
| R-squared | 0.11 | 0.12 | 0.12 | 0.12 | 0.12 |
| Log Likelihood | -2073.11 | -2067.34 | -2063.03 | -2067.22 | -2062.77 |

Notes: Standard errors two-way clustered by firm and year are reported in brackets.

+ p<0.10, * p<0.05, ** p<0.01, *** p<0.001. In constructing the dependent variables, we use residuals obtained from a regression model that pools observations across all years and includes firm fixed effects.

**Appendix E Table 8: Results of two-way fixed-effects regressions with D.V. estimated from models with industry fixed effects, using imprinted NEDs**

| D.V. | S.D. = 0.85, using industry fixed effects | | | | |
| --- | --- | --- | --- | --- | --- |
| Variables | Model 31 | Model 32 | Model 33 | Model 34 | Model 35 |
| Lambda | -2.202+ | -2.864* | -2.955* | -2.949* | -3.043* |
|  | [1.182] | [1.155] | [1.149] | [1.143] | [1.137] |
| Unabsorbed slack | 0.003 | 0.002 | 0.003 | 0.004 | 0.005 |
|  | [0.014] | [0.014] | [0.014] | [0.014] | [0.014] |
| HR slack | -1.866 | -2.104 | -2.165 | -2.368 | -2.432 |
|  | [2.335] | [2.333] | [2.319] | [2.325] | [2.311] |
| Absorbed slack | 0.010 | 0.004 | 0.000 | 0.009 | 0.005 |
|  | [0.144] | [0.144] | [0.144] | [0.144] | [0.143] |
| Performance | -0.002** | -0.002** | -0.002** | -0.002** | -0.002** |
|  | [0.001] | [0.001] | [0.001] | [0.001] | [0.001] |
| Log of firm age | 0.246* | 0.209+ | 0.212+ | 0.196 | 0.199 |
|  | [0.112] | [0.119] | [0.119] | [0.121] | [0.121] |
| Log of firm size | -0.170+ | -0.216* | -0.223* | -0.222* | -0.229* |
|  | [0.097] | [0.093] | [0.093] | [0.093] | [0.092] |
| Tenure | 0.005+ | 0.005 | 0.005 | 0.004 | 0.004 |
|  | [0.003] | [0.003] | [0.003] | [0.003] | [0.003] |
| CEO tenure | 0.001 | 0.001 | 0.001 | 0.001 | 0.001 |
|  | [0.002] | [0.002] | [0.002] | [0.002] | [0.002] |
| CEO tenure missing | -0.013 | -0.005 | -0.005 | -0.003 | -0.003 |
|  | [0.029] | [0.030] | [0.030] | [0.030] | [0.030] |
| Political background | -0.025 | -0.020 | -0.020 | -0.022 | -0.021 |
|  | [0.027] | [0.027] | [0.027] | [0.027] | [0.027] |
| Production background | 0.010 | 0.004 | 0.004 | 0.003 | 0.002 |
|  | [0.009] | [0.009] | [0.009] | [0.009] | [0.009] |
| NED | -0.023* | -0.025* | -0.023* | -0.026* | -0.024* |
|  | [0.010] | [0.009] | [0.009] | [0.009] | [0.009] |
| Cohort 1960–1969 NED | -0.008 | -0.016 | 0.001 | -0.017 | 0.001 |
|  | [0.010] | [0.010] | [0.015] | [0.010] | [0.015] |
| Activist shareholder | -0.059 | -0.059 | -0.059 | 0.224** | 0.224** |
|  | [0.067] | [0.068] | [0.068] | [0.068] | [0.068] |
| Cohort –1959 |  | -0.001 | 0.000 | 0.002 | 0.003 |
|  |  | [0.014] | [0.014] | [0.014] | [0.014] |
| Cohort 1960–1969 |  | 0.011* | 0.012* | 0.012* | 0.013** |
|  |  | [0.004] | [0.004] | [0.004] | [0.004] |
| Cohort 1970–1979 |  | 0.002 | 0.001 | 0.002 | 0.002 |
|  |  | [0.002] | [0.002] | [0.002] | [0.002] |
| Cohort 1980– |  | 0.002 | 0.002 | 0.002 | 0.002 |
|  |  | [0.005] | [0.005] | [0.006] | [0.006] |
| Cohort 1960–1969 |  |  | -0.005 |  | -0.005 |
| × Cohort 1960–1969 NED |  |  | [0.005] |  | [0.005] |
| Cohort 1960–1969 |  |  |  | -0.046*** | -0.046*** |
| × Activist shareholder |  |  |  | [0.009] | [0.009] |
| Constant | 2.566 | 3.660+ | 3.781+ | 3.836+ | 3.961* |
|  | [1.912] | [1.857] | [1.842] | [1.833] | [1.818] |
| Firm FE | Yes | Yes | Yes | Yes | Yes |
| Year FE | Yes | Yes | Yes | Yes | Yes |
| N | 5129 | 5129 | 5129 | 5129 | 5129 |
| R-squared | 0.41 | 0.41 | 0.41 | 0.41 | 0.41 |
| Log Likelihood | -1087.61 | -1081.55 | -1080.21 | -1070.30 | -1068.88 |

Notes: Standard errors two-way clustered by firm and year are reported in brackets.

+ p<0.10, * p<0.05, ** p<0.01, *** p<0.001. In constructing the dependent variables, we use residuals obtained from year-specific regression models that include one-digit Nikkei industry codes.

**Appendix E Table 9: Results of two-way fixed-effects regressions with D.V. estimated from models with firm fixed effects, using imprinted NEDs**

| D.V. | S.D. = 0.85, using firm fixed effects | | | | |
| --- | --- | --- | --- | --- | --- |
| Variables | Model 36 | Model 37 | Model 38 | Model 39 | Model 40 |
| Lambda | -1.140 | -1.740 | -1.895 | -1.808 | -1.966 |
|  | [1.715] | [1.664] | [1.666] | [1.653] | [1.654] |
| Unabsorbed slack | 0.005 | 0.004 | 0.006 | 0.006 | 0.007 |
|  | [0.021] | [0.021] | [0.021] | [0.021] | [0.021] |
| HR slack | -0.220 | -0.659 | -0.762 | -0.872 | -0.978 |
|  | [3.153] | [3.141] | [3.105] | [3.139] | [3.102] |
| Absorbed slack | 0.084 | 0.082 | 0.076 | 0.086 | 0.080 |
|  | [0.172] | [0.173] | [0.171] | [0.173] | [0.171] |
| Performance | 0.000 | 0.000 | 0.000 | 0.000 | 0.000 |
|  | [0.001] | [0.001] | [0.001] | [0.001] | [0.001] |
| Log of firm age | 0.164 | 0.125 | 0.129 | 0.114 | 0.119 |
|  | [0.173] | [0.176] | [0.176] | [0.176] | [0.176] |
| Log of firm size | -0.141 | -0.186 | -0.197 | -0.191 | -0.202 |
|  | [0.139] | [0.138] | [0.137] | [0.137] | [0.137] |
| Tenure | 0.001 | 0.002 | 0.002 | 0.001 | 0.001 |
|  | [0.005] | [0.005] | [0.005] | [0.005] | [0.005] |
| CEO tenure | 0.000 | 0.000 | 0.000 | 0.000 | 0.000 |
|  | [0.002] | [0.002] | [0.002] | [0.002] | [0.002] |
| CEO tenure missing | 0.015 | 0.023 | 0.023 | 0.025 | 0.025 |
|  | [0.042] | [0.041] | [0.041] | [0.041] | [0.041] |
| Political background | 0.020 | 0.023 | 0.024 | 0.022 | 0.023 |
|  | [0.039] | [0.039] | [0.039] | [0.039] | [0.039] |
| Production background | 0.001 | -0.002 | -0.002 | -0.003 | -0.003 |
|  | [0.011] | [0.010] | [0.011] | [0.010] | [0.011] |
| NED | -0.007 | -0.005 | -0.002 | -0.006 | -0.003 |
|  | [0.013] | [0.013] | [0.013] | [0.013] | [0.013] |
| Cohort 1960–1969 NED | -0.009 | -0.022 | 0.007 | -0.022 | 0.007 |
|  | [0.014] | [0.015] | [0.023] | [0.015] | [0.023] |
| Activist shareholder | -0.007 | -0.006 | -0.006 | 0.222** | 0.224** |
|  | [0.057] | [0.055] | [0.056] | [0.069] | [0.070] |
| Cohort –1959 |  | -0.023 | -0.022 | -0.021 | -0.019 |
|  |  | [0.016] | [0.016] | [0.016] | [0.015] |
| Cohort 1960–1969 |  | 0.011* | 0.013* | 0.012* | 0.014** |
|  |  | [0.005] | [0.005] | [0.005] | [0.005] |
| Cohort 1970–1979 |  | -0.002 | -0.002 | -0.002 | -0.002 |
|  |  | [0.004] | [0.004] | [0.004] | [0.004] |
| Cohort 1980– |  | 0.003 | 0.003 | 0.003 | 0.003 |
|  |  | [0.008] | [0.008] | [0.008] | [0.008] |
| Cohort 1960–1969 |  |  | -0.009 |  | -0.009 |
| × Cohort 1960–1969 NED |  |  | [0.005] |  | [0.005] |
| Cohort 1960–1969 |  |  |  | -0.037** | -0.037** |
| × Activist shareholder |  |  |  | [0.009] | [0.010] |
| Constant | 1.863 | 2.923 | 3.128 | 3.065 | 3.274 |
|  | [2.888] | [2.816] | [2.816] | [2.791] | [2.790] |
| Firm FE | Yes | Yes | Yes | Yes | Yes |
| Year FE | Yes | Yes | Yes | Yes | Yes |
| N | 5129 | 5129 | 5129 | 5129 | 5129 |
| R-squared | 0.11 | 0.12 | 0.12 | 0.12 | 0.12 |
| Log Likelihood | -2072.79 | -2065.72 | -2063.09 | -2060.72 | -2058.01 |

Notes: Standard errors two-way clustered by firm and year are reported in brackets.

+ p<0.10, * p<0.05, ** p<0.01, *** p<0.001. In constructing the dependent variables, we use residuals obtained from a regression model that pools observations across all years and includes firm fixed effects.

**Appendix E Table 10: Results of two-way fixed-effects regressions with D.V. estimated from models with industry fixed effects, using imprinted CEOs**

| D.V. | S.D. = 0.85, using industry fixed effects | | | |
| --- | --- | --- | --- | --- |
| Variables | Model 41 | Model 42 | Model 43 | Model 44 |
| Lambda | -2.359+ | -2.393+ | -2.347+ | -2.396+ |
|  | [1.187] | [1.187] | [1.187] | [1.188] |
| Unabsorbed slack | 0.003 | 0.004 | 0.002 | 0.003 |
|  | [0.014] | [0.013] | [0.014] | [0.013] |
| HR slack | -1.968 | -1.980 | -1.947 | -1.958 |
|  | [2.410] | [2.406] | [2.411] | [2.407] |
| Absorbed slack | -0.001 | 0.000 | -0.003 | -0.003 |
|  | [0.143] | [0.143] | [0.142] | [0.142] |
| Performance | -0.002** | -0.002** | -0.002** | -0.002** |
|  | [0.001] | [0.001] | [0.001] | [0.001] |
| Log of firm age | 0.234+ | 0.235+ | 0.236+ | 0.236+ |
|  | [0.113] | [0.113] | [0.113] | [0.113] |
| Log of firm size | -0.181+ | -0.186+ | -0.179+ | -0.187+ |
|  | [0.097] | [0.097] | [0.097] | [0.097] |
| Tenure | 0.005 | 0.005 | 0.005 | 0.005 |
|  | [0.003] | [0.003] | [0.003] | [0.003] |
| CEO tenure | 0.001 | 0.001 | 0.001 | 0.001 |
|  | [0.002] | [0.002] | [0.002] | [0.002] |
| CEO tenure missing | -0.009 | -0.010 | -0.008 | -0.009 |
|  | [0.030] | [0.030] | [0.030] | [0.030] |
| Political background | -0.024 | -0.023 | -0.024 | -0.023 |
|  | [0.027] | [0.027] | [0.027] | [0.027] |
| Production background | 0.010 | 0.010 | 0.010 | 0.010 |
|  | [0.009] | [0.009] | [0.009] | [0.009] |
| Cohort 1960–1969 NED | -0.008 | -0.008 | -0.008 | -0.008 |
|  | [0.010] | [0.010] | [0.010] | [0.010] |
| NED | -0.024* | -0.022* | -0.024* | -0.022* |
|  | [0.010] | [0.010] | [0.010] | [0.010] |
| Activist shareholder | -0.057 | -0.058 | -0.060 | -0.061 |
|  | [0.067] | [0.067] | [0.067] | [0.067] |
| Cohort –1959 CEO | -0.073 | -0.050 | -0.078+ | -0.043 |
|  | [0.043] | [0.047] | [0.044] | [0.044] |
| Cohort 1960–1969 CEO | 0.055+ | 0.058+ | 0.050 | 0.053+ |
|  | [0.030] | [0.028] | [0.030] | [0.029] |
| Cohort 1970–1979 CEO | -0.029 | -0.031 | -0.029 | -0.031 |
|  | [0.024] | [0.024] | [0.024] | [0.024] |
| Cohort 1980– CEO | 0.008 | 0.007 | 0.007 | 0.006 |
|  | [0.038] | [0.038] | [0.038] | [0.038] |
| Cohort 1960–1969 CEO |  | -0.037 |  | -0.056 |
| × NED |  | [0.040] |  | [0.034] |
| Cohort 1960–1969 CEO |  |  | 0.458* | 0.604** |
| × Activist shareholder |  |  | [0.208] | [0.190] |
| Constant | 2.860 | 2.943 | 2.829 | 2.946 |
|  | [1.914] | [1.912] | [1.916] | [1.909] |
| Firm FE | Yes | Yes | Yes | Yes |
| Year FE | Yes | Yes | Yes | Yes |
| N | 5129 | 5129 | 5129 | 5129 |
| R-squared | 0.41 | 0.41 | 0.41 | 0.41 |
| Log Likelihood | -1084.17 | -1082.97 | -1081.77 | -1079.15 |

Notes: Standard errors two-way clustered by firm and year are reported in brackets.

+ p<0.10, * p<0.05, ** p<0.01, *** p<0.001. In constructing the dependent variables, we use residuals obtained from year-specific regression models that include one-digit Nikkei industry codes.

**Appendix E Table 11: Results of two-way fixed-effects regressions with D.V. estimated from models with firm fixed effects, using imprinted CEOs**

| D.V. | S.D. = 0.85, using firm fixed effects | | | |
| --- | --- | --- | --- | --- |
| Variables | Model 45 | Model 46 | Model 47 | Model 48 |
| Lambda | -1.351 | -1.360 | -1.343 | -1.362 |
|  | [1.717] | [1.720] | [1.719] | [1.719] |
| Unabsorbed slack | 0.005 | 0.005 | 0.005 | 0.005 |
|  | [0.021] | [0.021] | [0.022] | [0.021] |
| HR slack | -0.395 | -0.398 | -0.381 | -0.385 |
|  | [3.238] | [3.235] | [3.236] | [3.231] |
| Absorbed slack | 0.064 | 0.064 | 0.062 | 0.062 |
|  | [0.169] | [0.169] | [0.168] | [0.168] |
| Performance | 0.000 | 0.000 | 0.000 | 0.000 |
|  | [0.001] | [0.001] | [0.001] | [0.001] |
| Log of firm age | 0.149 | 0.149 | 0.150 | 0.150 |
|  | [0.176] | [0.176] | [0.176] | [0.176] |
| Log of firm size | -0.157 | -0.158 | -0.155 | -0.158 |
|  | [0.139] | [0.140] | [0.139] | [0.140] |
| Tenure | 0.000 | 0.000 | 0.000 | 0.000 |
|  | [0.005] | [0.005] | [0.005] | [0.005] |
| CEO tenure | 0.000 | 0.000 | 0.000 | 0.000 |
|  | [0.002] | [0.002] | [0.002] | [0.002] |
| CEO tenure missing | 0.021 | 0.021 | 0.021 | 0.021 |
|  | [0.042] | [0.042] | [0.042] | [0.042] |
| Political background | 0.021 | 0.022 | 0.021 | 0.021 |
|  | [0.038] | [0.038] | [0.038] | [0.039] |
| Production background | 0.001 | 0.001 | 0.001 | 0.001 |
|  | [0.011] | [0.011] | [0.011] | [0.011] |
| Cohort 1960–1969 NED | -0.010 | -0.010 | -0.010 | -0.010 |
|  | [0.014] | [0.014] | [0.014] | [0.014] |
| NED | -0.008 | -0.008 | -0.008 | -0.007 |
|  | [0.013] | [0.013] | [0.013] | [0.013] |
| Activist shareholder | -0.005 | -0.006 | -0.007 | -0.007 |
|  | [0.057] | [0.057] | [0.057] | [0.057] |
| Cohort –1959 CEO | -0.035 | -0.029 | -0.038 | -0.025 |
|  | [0.082] | [0.079] | [0.083] | [0.079] |
| Cohort 1960–1969 CEO | 0.006 | 0.007 | 0.003 | 0.004 |
|  | [0.090] | [0.091] | [0.090] | [0.091] |
| Cohort 1970–1979 CEO | -0.042 | -0.043 | -0.042 | -0.043 |
|  | [0.028] | [0.028] | [0.028] | [0.028] |
| Cohort 1980– CEO | 0.015 | 0.015 | 0.015 | 0.014 |
|  | [0.045] | [0.045] | [0.045] | [0.045] |
| Cohort 1960–1969 CEO |  | -0.009 |  | -0.021 |
| × NED |  | [0.018] |  | [0.027] |
| Cohort 1960–1969 CEO |  |  | 0.299 | 0.353 |
| × Activist shareholder |  |  | [0.193] | [0.215] |
| Constant | 2.273 | 2.293 | 2.252 | 2.295 |
|  | [2.890] | [2.901] | [2.896] | [2.898] |
| Firm FE | Yes | Yes | Yes | Yes |
| Year FE | Yes | Yes | Yes | Yes |
| N | 5129 | 5129 | 5129 | 5129 |
| R-squared | 0.12 | 0.12 | 0.12 | 0.12 |
| Log Likelihood | -2068.15 | -2068.10 | -2067.45 | -2067.21 |

Notes: Standard errors two-way clustered by firm and year are reported in brackets.

+ p<0.10, * p<0.05, ** p<0.01, *** p<0.001. In constructing the dependent variables, we use residuals obtained from a regression model that pools observations across all years and includes firm fixed effects.

**Appendix E Table 12: Results of two-way fixed-effects logistic regressions with D.V. estimated from models with industry fixed effects**

| D.V. | S.D. = 0.85, using industry fixed effects | | | |
| --- | --- | --- | --- | --- |
| Variables | Model 49 | Model 50 | Model 51 | Model 52 |
| Lambda | -23.072* | -23.809* | -23.651* | -24.314* |
|  | [10.189] | [10.272] | [10.245] | [10.315] |
| Unabsorbed slack | -0.006 | 0.032 | 0.034 | 0.064 |
|  | [0.154] | [0.156] | [0.155] | [0.157] |
| HR slack | -21.868 | -21.119 | -23.659 | -22.827 |
|  | [18.931] | [18.929] | [18.943] | [18.943] |
| Absorbed slack | -0.097 | -0.127 | -0.084 | -0.107 |
|  | [1.145] | [1.143] | [1.150] | [1.147] |
| Performance | -0.023** | -0.022** | -0.025*** | -0.024** |
|  | [0.007] | [0.007] | [0.007] | [0.007] |
| Log of firm age | 1.597+ | 1.518+ | 1.486+ | 1.428 |
|  | [0.887] | [0.882] | [0.878] | [0.875] |
| Log of firm size | -1.779* | -1.900* | -1.834* | -1.944* |
|  | [0.770] | [0.774] | [0.774] | [0.778] |
| Tenure | 0.044 | 0.044 | 0.037 | 0.036 |
|  | [0.028] | [0.028] | [0.028] | [0.028] |
| CEO tenure | 0.010 | 0.013 | 0.009 | 0.011 |
|  | [0.014] | [0.015] | [0.015] | [0.015] |
| CEO tenure missing | -0.028 | -0.063 | -0.018 | -0.051 |
|  | [0.260] | [0.260] | [0.261] | [0.261] |
| Political background | -0.168 | -0.217 | -0.207 | -0.256 |
|  | [0.294] | [0.296] | [0.301] | [0.303] |
| Production background | 0.052 | 0.047 | 0.032 | 0.029 |
|  | [0.079] | [0.079] | [0.079] | [0.079] |
| Cohort 1960–1969 NED | -0.190+ | -0.110 | -0.220* | -0.142 |
|  | [0.110] | [0.116] | [0.111] | [0.117] |
| NED | -0.269** | -0.093 | -0.276** | -0.116 |
|  | [0.084] | [0.102] | [0.085] | [0.103] |
| Activist shareholder | -0.392+ | -0.441* | 2.335* | 1.925* |
|  | [0.202] | [0.208] | [0.947] | [0.932] |
| Cohort –1959 | -0.006 | 0.041 | 0.026 | 0.067 |
|  | [0.114] | [0.117] | [0.115] | [0.117] |
| Cohort 1960–1969 | 0.126*** | 0.157*** | 0.140*** | 0.167*** |
|  | [0.036] | [0.038] | [0.037] | [0.038] |
| Cohort 1970–1979 | 0.017 | 0.014 | 0.021 | 0.018 |
|  | [0.030] | [0.030] | [0.029] | [0.030] |
| Cohort 1980– | 0.009 | 0.002 | 0.009 | 0.003 |
|  | [0.053] | [0.053] | [0.053] | [0.053] |
| Cohort 1960–1969 |  | -0.060** |  | -0.056** |
| × NED |  | [0.021] |  | [0.021] |
| Cohort 1960–1969 |  |  | -0.531* | -0.437* |
| × Activist shareholder |  |  | [0.225] | [0.221] |
| Firm FE | Yes | Yes | Yes | Yes |
| Year FE | Yes | Yes | Yes | Yes |
| N | 3153 | 3153 | 3153 | 3153 |
| Log Likelihood | -1086.80 | -1082.06 | -1079.29 | -1075.44 |

Notes: Robust standard errors are in brackets. + p<0.10, * p<0.05, ** p<0.01, *** p<0.001. In constructing the dependent variables, we use residuals obtained from year-specific regression models that include one-digit Nikkei industry codes.

**Appendix E Table 13: Results of two-way fixed-effects logistic regressions with D.V. estimated from models with firm fixed effects**

| D.V. | S.D. = 0.85, using firm fixed effects | | | |
| --- | --- | --- | --- | --- |
| Variables | Model 53 | Model 54 | Model 55 | Model 56 |
| Lambda | -5.901 | -6.343 | -6.329 | -6.700 |
|  | [7.760] | [7.789] | [7.771] | [7.796] |
| Unabsorbed slack | 0.044 | 0.063 | 0.059 | 0.075 |
|  | [0.115] | [0.116] | [0.116] | [0.116] |
| HR slack | -2.856 | -2.953 | -4.741 | -4.684 |
|  | [15.480] | [15.528] | [15.511] | [15.553] |
| Absorbed slack | 0.600 | 0.487 | 0.626 | 0.521 |
|  | [0.983] | [0.982] | [0.986] | [0.985] |
| Performance | 0.000 | 0.001 | -0.001 | 0.000 |
|  | [0.006] | [0.006] | [0.006] | [0.006] |
| Log of firm age | 0.876 | 0.818 | 0.783 | 0.739 |
|  | [0.775] | [0.777] | [0.770] | [0.773] |
| Log of firm size | -0.874 | -0.927 | -0.911 | -0.956 |
|  | [0.597] | [0.599] | [0.598] | [0.600] |
| Tenure | 0.012 | 0.010 | 0.008 | 0.006 |
|  | [0.022] | [0.022] | [0.022] | [0.023] |
| CEO tenure | -0.001 | 0.001 | -0.001 | 0.001 |
|  | [0.012] | [0.012] | [0.012] | [0.012] |
| CEO tenure missing | 0.155 | 0.133 | 0.171 | 0.148 |
|  | [0.205] | [0.206] | [0.205] | [0.206] |
| Political background | 0.228 | 0.206 | 0.224 | 0.201 |
|  | [0.223] | [0.224] | [0.224] | [0.225] |
| Production background | -0.013 | -0.013 | -0.022 | -0.022 |
|  | [0.062] | [0.062] | [0.062] | [0.062] |
| Cohort 1960–1969 NED | -0.153+ | -0.084 | -0.166+ | -0.099 |
|  | [0.089] | [0.093] | [0.089] | [0.094] |
| NED | -0.029 | 0.109 | -0.032 | 0.098 |
|  | [0.067] | [0.080] | [0.067] | [0.081] |
| Activist shareholder | -0.008 | -0.044 | 1.921* | 1.636* |
|  | [0.188] | [0.191] | [0.848] | [0.778] |
| Cohort –1959 | -0.156 | -0.109 | -0.137 | -0.095 |
|  | [0.104] | [0.105] | [0.104] | [0.105] |
| Cohort 1960–1969 | 0.073** | 0.096** | 0.080** | 0.100*** |
|  | [0.028] | [0.029] | [0.028] | [0.030] |
| Cohort 1970–1979 | -0.015 | -0.020 | -0.013 | -0.018 |
|  | [0.023] | [0.023] | [0.023] | [0.023] |
| Cohort 1980– | 0.021 | 0.011 | 0.022 | 0.013 |
|  | [0.042] | [0.042] | [0.042] | [0.042] |
| Cohort 1960–1969 |  | -0.050** |  | -0.047** |
| × NED |  | [0.017] |  | [0.017] |
| Cohort 1960–1969 |  |  | -0.367+ | -0.301 |
| × Activist shareholder |  |  | [0.214] | [0.188] |
| Firm FE | Yes | Yes | Yes | Yes |
| Year FE | Yes | Yes | Yes | Yes |
| N | 4654 | 4654 | 4654 | 4654 |
| Log Likelihood | -1641.80 | -1637.21 | -1637.14 | -1633.19 |

Notes: Robust standard errors are in brackets. + p<0.10, * p<0.05, ** p<0.01, *** p<0.001. In constructing the dependent variables, we use residuals obtained from a regression model that pools observations across all years and includes firm fixed effects.

**Appendix E Table 14: Results of two-way fixed-effects regressions with D.V. estimated from models with industry fixed effects**

| D.V. | Raw Score, using industry fixed effects | | | |
| --- | --- | --- | --- | --- |
| Variables | Model 57 | Model 58 | Model 59 | Model 60 |
| Lambda | -8.255+ | -8.368+ | -8.540+ | -8.639+ |
|  | [4.520] | [4.538] | [4.475] | [4.492] |
| Unabsorbed slack | 0.002 | 0.009 | 0.010 | 0.016 |
|  | [0.044] | [0.044] | [0.044] | [0.044] |
| HR slack | -8.550 | -8.509 | -9.441 | -9.384 |
|  | [8.152] | [8.151] | [8.137] | [8.139] |
| Absorbed slack | 0.041 | -0.002 | 0.058 | 0.017 |
|  | [0.394] | [0.394] | [0.391] | [0.393] |
| Performance | -0.002 | -0.002 | -0.003 | -0.003 |
|  | [0.003] | [0.003] | [0.003] | [0.003] |
| Log of firm age | 0.759+ | 0.740+ | 0.716+ | 0.700+ |
|  | [0.404] | [0.402] | [0.401] | [0.399] |
| Log of firm size | -0.600 | -0.609 | -0.619 | -0.628 |
|  | [0.383] | [0.382] | [0.380] | [0.379] |
| Tenure | 0.019 | 0.019 | 0.016 | 0.016 |
|  | [0.011] | [0.011] | [0.011] | [0.011] |
| CEO tenure | 0.005 | 0.005 | 0.005 | 0.005 |
|  | [0.005] | [0.005] | [0.006] | [0.005] |
| CEO tenure missing | -0.081 | -0.085 | -0.073 | -0.077 |
|  | [0.102] | [0.104] | [0.102] | [0.103] |
| Political background | -0.061 | -0.066 | -0.065 | -0.069 |
|  | [0.089] | [0.091] | [0.088] | [0.089] |
| Production background | 0.002 | 0.002 | -0.003 | -0.003 |
|  | [0.025] | [0.025] | [0.025] | [0.025] |
| Cohort 1960–1969 NED | -0.039 | -0.016 | -0.042 | -0.021 |
|  | [0.035] | [0.037] | [0.034] | [0.036] |
| NED | -0.041 | 0.001 | -0.045 | -0.005 |
|  | [0.030] | [0.034] | [0.030] | [0.034] |
| Activist shareholder | -0.150 | -0.158 | 0.804** | 0.777** |
|  | [0.240] | [0.246] | [0.200] | [0.209] |
| Cohort –1959 | -0.027 | -0.012 | -0.017 | -0.003 |
|  | [0.041] | [0.041] | [0.041] | [0.041] |
| Cohort 1960–1969 | 0.033* | 0.042** | 0.035* | 0.043** |
|  | [0.013] | [0.014] | [0.013] | [0.014] |
| Cohort 1970–1979 | 0.003 | 0.002 | 0.004 | 0.003 |
|  | [0.008] | [0.008] | [0.008] | [0.008] |
| Cohort 1980– | 0.006 | 0.003 | 0.006 | 0.003 |
|  | [0.018] | [0.018] | [0.018] | [0.018] |
| Cohort 1960–1969 |  | -0.016+ |  | -0.015+ |
| × NED |  | [0.008] |  | [0.008] |
| Cohort 1960–1969 |  |  | -0.154*** | -0.150*** |
| × Activist shareholder |  |  | [0.026] | [0.027] |
| Constant | 9.727 | 9.953 | 10.319 | 10.519 |
|  | [7.583] | [7.578] | [7.501] | [7.502] |
| Firm FE | Yes | Yes | Yes | Yes |
| Year FE | Yes | Yes | Yes | Yes |
| N | 5129 | 5129 | 5129 | 5129 |
| R-squared | 0.52 | 0.52 | 0.52 | 0.52 |
| Log Likelihood | -6452.94 | -6447.96 | -6437.17 | -6432.80 |

Notes: Standard errors two-way clustered by firm and year are reported in brackets.

+ p<0.10, * p<0.05, ** p<0.01, *** p<0.001. In constructing the dependent variables, we use residuals obtained from year-specific regression models that include one-digit Nikkei industry codes.

**Appendix E Table 15: Results of two-way fixed-effects regressions with D.V. estimated from models with firm fixed effects**

| D.V. | Raw Score, using firm fixed effects | | | |
| --- | --- | --- | --- | --- |
| Variables | Model 61 | Model 62 | Model 63 | Model 64 |
| Lambda | -5.167 | -5.259 | -5.341 | -5.424 |
|  | [3.947] | [3.917] | [3.911] | [3.884] |
| Unabsorbed slack | -0.021 | -0.015 | -0.017 | -0.011 |
|  | [0.043] | [0.043] | [0.044] | [0.044] |
| HR slack | -1.305 | -1.271 | -1.851 | -1.803 |
|  | [7.300] | [7.253] | [7.295] | [7.249] |
| Absorbed slack | -0.105 | -0.141 | -0.095 | -0.129 |
|  | [0.381] | [0.378] | [0.380] | [0.377] |
| Performance | -0.002 | -0.002 | -0.002 | -0.002 |
|  | [0.002] | [0.002] | [0.002] | [0.002] |
| Log of firm age | 0.207 | 0.191 | 0.181 | 0.167 |
|  | [0.368] | [0.365] | [0.366] | [0.363] |
| Log of firm size | -0.499 | -0.507 | -0.512 | -0.519 |
|  | [0.332] | [0.330] | [0.329] | [0.328] |
| Tenure | 0.006 | 0.006 | 0.004 | 0.004 |
|  | [0.011] | [0.011] | [0.011] | [0.011] |
| CEO tenure | 0.001 | 0.002 | 0.001 | 0.002 |
|  | [0.004] | [0.004] | [0.004] | [0.004] |
| CEO tenure missing | -0.015 | -0.019 | -0.011 | -0.014 |
|  | [0.096] | [0.096] | [0.096] | [0.096] |
| Political background | 0.021 | 0.017 | 0.019 | 0.015 |
|  | [0.076] | [0.077] | [0.075] | [0.076] |
| Production background | -0.019 | -0.019 | -0.022 | -0.022 |
|  | [0.020] | [0.020] | [0.021] | [0.020] |
| Cohort 1960–1969 NED | -0.024 | -0.005 | -0.026 | -0.008 |
|  | [0.032] | [0.034] | [0.032] | [0.034] |
| NED | -0.031 | 0.004 | -0.033 | 0.000 |
|  | [0.028] | [0.029] | [0.028] | [0.028] |
| Activist shareholder | 0.048 | 0.041 | 0.632** | 0.610** |
|  | [0.151] | [0.156] | [0.187] | [0.193] |
| Cohort –1959 | -0.046 | -0.034 | -0.040 | -0.028 |
|  | [0.033] | [0.032] | [0.033] | [0.032] |
| Cohort 1960–1969 | 0.023* | 0.030* | 0.025* | 0.032* |
|  | [0.010] | [0.011] | [0.010] | [0.011] |
| Cohort 1970–1979 | -0.003 | -0.004 | -0.002 | -0.003 |
|  | [0.008] | [0.008] | [0.008] | [0.008] |
| Cohort 1980– | 0.012 | 0.010 | 0.012 | 0.010 |
|  | [0.017] | [0.017] | [0.017] | [0.017] |
| Cohort 1960–1969 |  | -0.013+ |  | -0.012+ |
| × NED |  | [0.006] |  | [0.006] |
| Cohort 1960–1969 |  |  | -0.094** | -0.092** |
| × Activist shareholder |  |  | [0.024] | [0.025] |
| Constant | 8.819 | 9.005 | 9.182 | 9.349 |
|  | [6.738] | [6.694] | [6.670] | [6.633] |
| Firm FE | Yes | Yes | Yes | Yes |
| Year FE | Yes | Yes | Yes | Yes |
| N | 5129 | 5129 | 5129 | 5129 |
| R-squared | 0.12 | 0.12 | 0.12 | 0.12 |
| Log Likelihood | -5477.43 | -5472.52 | -5468.77 | -5464.32 |

Notes: Standard errors two-way clustered by firm and year are reported in brackets.

+ p<0.10, * p<0.05, ** p<0.01, *** p<0.001. In constructing the dependent variables, we use residuals obtained from a regression model that pools observations across all years and includes firm fixed effects.

**Appendix E Table 16: Results of two-way fixed-effects regressions with D.V. estimated from models with industry fixed effects, using the alternative thresholds**

| D.V. | S.D. = 0.70, using industry fixed effects | | | | S.D. = 1.00, using industry fixed effects | | | |
| --- | --- | --- | --- | --- | --- | --- | --- | --- |
| Variable | Model 65 | Model 66 | Model 67 | Model 68 | Model 69 | Model 70 | Model 71 | Model 72 |
| Lambda | -2.992* | -3.040* | -3.075* | -3.118* | -2.563+ | -2.601+ | -2.649+ | -2.683+ |
|  | [1.191] | [1.207] | [1.183] | [1.198] | [1.297] | [1.308] | [1.281] | [1.292] |
| Unabsorbed slack | -0.005 | -0.002 | -0.003 | 0.000 | 0.011 | 0.013 | 0.013 | 0.015 |
|  | [0.013] | [0.013] | [0.013] | [0.013] | [0.014] | [0.014] | [0.014] | [0.014] |
| HR slack | -2.162 | -2.145 | -2.420 | -2.395 | -2.886 | -2.871 | -3.153 | -3.134 |
|  | [2.455] | [2.435] | [2.447] | [2.428] | [2.347] | [2.348] | [2.340] | [2.341] |
| Absorbed slack | 0.018 | 0.000 | 0.023 | 0.005 | 0.015 | 0.000 | 0.020 | 0.006 |
|  | [0.155] | [0.153] | [0.154] | [0.153] | [0.150] | [0.150] | [0.149] | [0.149] |
| Performance | -0.002* | -0.002* | -0.002* | -0.002* | -0.001+ | -0.001 | -0.001+ | -0.001+ |
|  | [0.001] | [0.001] | [0.001] | [0.001] | [0.001] | [0.001] | [0.001] | [0.001] |
| Log of firm age | 0.228+ | 0.220+ | 0.216 | 0.208 | 0.205+ | 0.199+ | 0.192+ | 0.187 |
|  | [0.127] | [0.124] | [0.129] | [0.126] | [0.109] | [0.108] | [0.109] | [0.108] |
| Log of firm size | -0.231* | -0.235* | -0.236* | -0.240* | -0.184 | -0.187 | -0.190+ | -0.193+ |
|  | [0.090] | [0.090] | [0.090] | [0.090] | [0.108] | [0.108] | [0.107] | [0.107] |
| Tenure | 0.004 | 0.004 | 0.003 | 0.003 | 0.004 | 0.004 | 0.004 | 0.003 |
|  | [0.003] | [0.003] | [0.004] | [0.003] | [0.003] | [0.003] | [0.004] | [0.004] |
| CEO tenure | 0.001 | 0.001 | 0.001 | 0.001 | 0.001 | 0.001 | 0.001 | 0.001 |
|  | [0.002] | [0.002] | [0.002] | [0.002] | [0.002] | [0.002] | [0.002] | [0.002] |
| CEO tenure missing | -0.006 | -0.008 | -0.004 | -0.006 | 0.001 | 0.000 | 0.004 | 0.002 |
|  | [0.028] | [0.029] | [0.028] | [0.029] | [0.032] | [0.032] | [0.031] | [0.032] |
| Political background | -0.013 | -0.015 | -0.014 | -0.016 | -0.012 | -0.014 | -0.013 | -0.015 |
|  | [0.031] | [0.031] | [0.031] | [0.032] | [0.024] | [0.024] | [0.024] | [0.024] |
| Production background | 0.004 | 0.004 | 0.003 | 0.003 | -0.001 | -0.001 | -0.002 | -0.002 |
|  | [0.010] | [0.010] | [0.010] | [0.010] | [0.010] | [0.010] | [0.010] | [0.010] |
| Cohort 1960–1969 NED | -0.014 | -0.004 | -0.015 | -0.006 | -0.019+ | -0.011 | -0.020+ | -0.013 |
|  | [0.011] | [0.012] | [0.011] | [0.012] | [0.010] | [0.011] | [0.009] | [0.010] |
| NED | -0.026* | -0.008 | -0.027* | -0.009 | -0.017+ | -0.002 | -0.018+ | -0.004 |
|  | [0.010] | [0.011] | [0.010] | [0.011] | [0.009] | [0.009] | [0.009] | [0.009] |
| Activist shareholder | -0.065 | -0.068 | 0.211** | 0.200* | -0.053 | -0.055 | 0.234** | 0.225** |
|  | [0.065] | [0.066] | [0.069] | [0.068] | [0.072] | [0.074] | [0.073] | [0.075] |
| Cohort –1959 | -0.004 | 0.003 | -0.001 | 0.005 | 0.002 | 0.007 | 0.005 | 0.010 |
|  | [0.014] | [0.014] | [0.014] | [0.014] | [0.014] | [0.014] | [0.014] | [0.014] |
| Cohort 1960–1969 | 0.012* | 0.015** | 0.012* | 0.016** | 0.009* | 0.012* | 0.010* | 0.013* |
|  | [0.005] | [0.005] | [0.005] | [0.005] | [0.004] | [0.004] | [0.004] | [0.005] |
| Cohort 1970–1979 | 0.000 | 0.000 | 0.001 | 0.000 | -0.002 | -0.002 | -0.001 | -0.002 |
|  | [0.003] | [0.003] | [0.003] | [0.003] | [0.003] | [0.003] | [0.003] | [0.003] |
| Cohort 1980– | 0.002 | 0.000 | 0.002 | 0.000 | -0.002 | -0.003 | -0.002 | -0.003 |
|  | [0.006] | [0.006] | [0.006] | [0.006] | [0.005] | [0.005] | [0.005] | [0.005] |
| Cohort 1960–1969 |  | -0.007* |  | -0.006* |  | -0.005* |  | -0.005+ |
| × NED |  | [0.003] |  | [0.003] |  | [0.003] |  | [0.002] |
| Cohort 1960–1969 |  |  | -0.044*** | -0.043*** |  |  | -0.046*** | -0.045*** |
| × Activist shareholder |  |  | [0.009] | [0.009] |  |  | [0.009] | [0.009] |
| Constant | 3.866+ | 3.961+ | 4.037* | 4.123* | 3.074 | 3.152 | 3.252 | 3.321 |
|  | [1.893] | [1.915] | [1.875] | [1.899] | [2.109] | [2.107] | [2.081] | [2.082] |
| Firm FE | Yes | Yes | Yes | Yes | Yes | Yes | Yes | Yes |
| Year FE | Yes | Yes | Yes | Yes | Yes | Yes | Yes | Yes |
| N | 5129 | 5129 | 5129 | 5129 | 5129 | 5129 | 5129 | 5129 |
| R-squared | 0.40 | 0.41 | 0.41 | 0.41 | 0.39 | 0.39 | 0.40 | 0.40 |
| Log Likelihood | -1203.60 | -1196.68 | -1193.39 | -1187.06 | -643.65 | -638.01 | -629.93 | -624.91 |

Notes: Standard errors two-way clustered by firm and year are reported in brackets.

+ p<0.10, * p<0.05, ** p<0.01, *** p<0.001. In constructing the dependent variables, we use residuals obtained from year-specific regression models that include one-digit Nikkei industry codes.

**Appendix E Table 17: Results of two-way fixed-effects regressions with D.V. estimated from models with firm fixed effects, using the alternative thresholds**

| D.V. | S.D.= 0.70, using firm fixed effects | | | | S.D.= 1.00, using firm fixed effects | | | |
| --- | --- | --- | --- | --- | --- | --- | --- | --- |
| Variable | Model 73 | Model 74 | Model 75 | Model 76 | Model 77 | Model 78 | Model 79 | Model 80 |
| Lambda | -1.855 | -1.912 | -1.926 | -1.978 | -2.183 | -2.221 | -2.243 | -2.278 |
|  | [1.988] | [1.941] | [1.980] | [1.935] | [1.375] | [1.386] | [1.363] | [1.374] |
| Unabsorbed slack | 0.002 | 0.006 | 0.004 | 0.008 | 0.001 | 0.003 | 0.002 | 0.005 |
|  | [0.023] | [0.023] | [0.023] | [0.023] | [0.019] | [0.019] | [0.019] | [0.019] |
| HR slack | -1.295 | -1.274 | -1.516 | -1.486 | -0.536 | -0.522 | -0.724 | -0.705 |
|  | [3.506] | [3.469] | [3.499] | [3.462] | [3.039] | [3.008] | [3.045] | [3.015] |
| Absorbed slack | 0.110 | 0.088 | 0.114 | 0.093 | 0.042 | 0.027 | 0.046 | 0.031 |
|  | [0.182] | [0.178] | [0.182] | [0.178] | [0.143] | [0.141] | [0.142] | [0.140] |
| Performance | -0.001 | -0.001 | -0.001 | -0.001 | 0.001 | 0.001 | 0.000 | 0.000 |
|  | [0.001] | [0.001] | [0.001] | [0.001] | [0.001] | [0.001] | [0.001] | [0.001] |
| Log of firm age | 0.126 | 0.116 | 0.115 | 0.106 | 0.164 | 0.157 | 0.155 | 0.149 |
|  | [0.207] | [0.204] | [0.206] | [0.204] | [0.167] | [0.165] | [0.167] | [0.165] |
| Log of firm size | -0.190 | -0.195 | -0.195 | -0.200 | -0.213+ | -0.216+ | -0.217+ | -0.220+ |
|  | [0.160] | [0.159] | [0.160] | [0.158] | [0.119] | [0.119] | [0.118] | [0.118] |
| Tenure | 0.006 | 0.006 | 0.005 | 0.005 | 0.001 | 0.001 | 0.001 | 0.001 |
|  | [0.005] | [0.005] | [0.005] | [0.005] | [0.004] | [0.004] | [0.004] | [0.004] |
| CEO tenure | 0.001 | 0.001 | 0.001 | 0.001 | 0.001 | 0.001 | 0.001 | 0.001 |
|  | [0.002] | [0.002] | [0.002] | [0.002] | [0.002] | [0.002] | [0.002] | [0.002] |
| CEO tenure missing | 0.013 | 0.011 | 0.015 | 0.013 | -0.005 | -0.006 | -0.003 | -0.005 |
|  | [0.047] | [0.047] | [0.047] | [0.047] | [0.037] | [0.037] | [0.037] | [0.037] |
| Political background | 0.014 | 0.012 | 0.013 | 0.011 | 0.008 | 0.006 | 0.007 | 0.006 |
|  | [0.046] | [0.046] | [0.046] | [0.046] | [0.035] | [0.035] | [0.034] | [0.035] |
| Production background | 0.003 | 0.003 | 0.002 | 0.002 | -0.002 | -0.002 | -0.003 | -0.003 |
|  | [0.013] | [0.013] | [0.013] | [0.013] | [0.008] | [0.008] | [0.008] | [0.008] |
| Cohort 1960–1969 NED | -0.017 | -0.005 | -0.018 | -0.006 | -0.014 | -0.006 | -0.014 | -0.007 |
|  | [0.017] | [0.017] | [0.017] | [0.017] | [0.013] | [0.015] | [0.013] | [0.014] |
| NED | -0.005 | 0.016 | -0.006 | 0.015 | -0.012 | 0.003 | -0.012 | 0.001 |
|  | [0.013] | [0.014] | [0.013] | [0.014] | [0.011] | [0.013] | [0.011] | [0.013] |
| Activist shareholder | -0.005 | -0.009 | 0.231** | 0.217** | 0.008 | 0.006 | 0.211** | 0.201* |
|  | [0.056] | [0.059] | [0.071] | [0.072] | [0.052] | [0.053] | [0.069] | [0.069] |
| Cohort –1959 | -0.027 | -0.019 | -0.024 | -0.017 | -0.024+ | -0.019 | -0.022 | -0.017 |
|  | [0.018] | [0.018] | [0.018] | [0.018] | [0.013] | [0.012] | [0.013] | [0.012] |
| Cohort 1960–1969 | 0.006 | 0.011+ | 0.007 | 0.011+ | 0.012* | 0.015** | 0.012* | 0.015** |
|  | [0.005] | [0.005] | [0.005] | [0.005] | [0.005] | [0.004] | [0.005] | [0.004] |
| Cohort 1970–1979 | -0.002 | -0.003 | -0.002 | -0.002 | -0.002 | -0.002 | -0.001 | -0.002 |
|  | [0.004] | [0.004] | [0.004] | [0.004] | [0.004] | [0.004] | [0.004] | [0.004] |
| Cohort 1980– | 0.009 | 0.007 | 0.009 | 0.007 | 0.003 | 0.002 | 0.003 | 0.002 |
|  | [0.010] | [0.009] | [0.010] | [0.010] | [0.007] | [0.007] | [0.007] | [0.007] |
| Cohort 1960–1969 |  | -0.008** |  | -0.008** |  | -0.005+ |  | -0.005+ |
| × NED |  | [0.003] |  | [0.002] |  | [0.003] |  | [0.003] |
| Cohort 1960–1969 |  |  | -0.038** | -0.036** |  |  | -0.033** | -0.031** |
| × Activist shareholder |  |  | [0.010] | [0.010] |  |  | [0.009] | [0.008] |
| Constant | 3.069 | 3.184 | 3.216 | 3.321 | 3.338 | 3.414 | 3.463 | 3.533 |
|  | [3.241] | [3.187] | [3.221] | [3.170] | [2.361] | [2.367] | [2.338] | [2.345] |
| Firm FE | Yes | Yes | Yes | Yes | Yes | Yes | Yes | Yes |
| Year FE | Yes | Yes | Yes | Yes | Yes | Yes | Yes | Yes |
| N | 5129 | 5129 | 5129 | 5129 | 5129 | 5129 | 5129 | 5129 |
| R-squared | 0.11 | 0.11 | 0.11 | 0.12 | 0.13 | 0.13 | 0.13 | 0.13 |
| Log Likelihood | -2474.22 | -2468.12 | -2469.66 | -2463.94 | -1610.91 | -1607.15 | -1606.23 | -1602.78 |

Notes: Standard errors two-way clustered by firm and year are reported in brackets.

+ p<0.10, * p<0.05, ** p<0.01, *** p<0.001. In constructing the dependent variables, we use residuals obtained from a regression model that pools observations across all years and includes firm fixed effects.

**Works cited**

Environment Agency (1973) Kankyō Hakusho: Shōwa 48-nenban [White paper on the environment, 1973] (in Japanese). Tokyo: Environment Agency.

Niigata Prefecture (2024) ‘Niigata-Ken no Kankyō: Genjō to Taisaku [The Environment of Niigata Prefecture: Current Conditions and Measures’] (in Japanese).

Olcott, G. (2009) Conflict and Change: Foreign Ownership and The Japanese Firm. Cambridge: Cambridge University Press.

Prime Minister’s Office (1971) Kōgai Hakusho: Shōwa 46-nenban [White paper on environmental pollution, 1971] (in Japanese). Tokyo: Prime Minister’s Office.
