# Supplementary material for: Clinical outcomes and quality of life after proton therapy for cranial and extracranial solitary fibrous tumors
Source: BJR Open. 2026 Oct 26;8(1):tzag023. doi: 10.1093/bjro/tzag023 (PMC13730268; doi:10.1093/bjro/tzag023)
Supplement: Supplementary file 1 [file tzag023_supplementary_data.zip › Supplementary_Data/Supplementary Material.docx]

**Supplementary Material**

Supplementary Figure 1: Kaplan–Meier analysis of local control stratified by tumor localization and recurrence status.
